# Radiation induces senescence in lymphatic endothelial cells (LECs) and murine tail lymphedema tissue, contributing to lymphedema progression

**DOI:** 10.1038/s41419-026-08955-z

**Published:** 2026-06-10

**Authors:** Samaneh Safarpour, Karina P. Gomes, Jacob Korodimas, Milica Vignjevic, Madhumita S. Manivannan, Nirav Patel, Xiaoyan Yang, Spencer B. Gibson

**Affiliations:** 1 Department of Oncology, Faculty of Medicine and Dentistry, University of Alberta, Edmonton, AB Canada; 2 Department of Oncology, Kipnes Endowed Chair in Lymphatic Disorders University of Alberta, Edmonton, AB Canada

**Keywords:** Apoptosis, Senescence

## Abstract

Cancer-related lymphedema (CRL) is an incurable disease characterized by progressive swelling of extremities. One of the risk factors in developing CRL is cancer treatments, including surgery and radiation. This leads to damage to the lymphatic system, causing accumulation of interstitial fluid, infiltration of inflammatory cells and cytokine release, tissue remodeling, accumulation of subcutaneous fat, and fibrosis. Radiation therapy (RT) inhibits lymphatic proliferation and survival by downregulating vascular endothelial growth factor receptor 3 (VEGFR-3) in lymphatic endothelial cells (LECs). How radiation affects CRL progression remains unclear. In this study, we found that RT reduced LEC growth and increased senescence in LECs as determined by increased senescence-associated β-galactosidase (SA-βgal) activity and increased protein expression of senescence markers p53, p21, and p16. Using the mouse tail lymphedema model, we found that tail swelling was increased after RT in both sham control and lymphatic ablation mice. After 30 days, tail swelling was maintained in RT treated mice with lymphatic ablation, whereas RT treated sham controls showed reduced swelling. This corresponded to an increase in senescent cells and apoptosis after RT in lymphatic-ablated mice compared to sham controls. We also found increased levels of senescence-related cytokines and chemokines in lymphedema patients’ plasma samples who had RT compared to surgery alone or non-lymphedema controls. Finally, we found that RT increased anti-apoptotic protein BCL-2 in human LECs and lymphatic ablated mouse tissue. Treatment with the senolytic agent venetoclax (VCX), a BCL-2 inhibitor, selectively killed RT-induced senescent LECs and reduced swelling in the RT-induced tail lymphedema model. This suggests that RT contributes to CRL, at least in part, by inducing cellular senescence.

## Introduction

Cancer survivorship is increasing; in 2022, 70% of cancer survivors had lived 5 years or more after diagnosis; however, they often develop long-term toxicities due to these therapies [1, 2]. One of the major toxicities is cancer-related lymphedema (CRL) affecting up to 50% of cancer survivors [3, 4]. CRL is a debilitating disease due to damage to the lymphatic system, leading to the accumulation of extracellular fluid in the extremities of patients [5]. Changes in lymphatic flow are often followed by inflammation and cytokine release, tissue remodeling, accumulation of subcutaneous fat, and fibrosis [5–7]. Patients experience pain, lack of mobility, infections, and decreased quality of life [8]. Lymphedema is a clinical burden with no effective treatments, and the available treatments, such as compression bandages, only address the symptoms [9–11]. In CRL, risk factors for lymphedema are high body mass index, node-positive diseases, and extensive treatments such as lymph node dissection, radiation therapy (RT), and/or chemotherapy [12–16]. Specifically, RT significantly elevates the risk of lymphedema in both upper and lower extremities. In breast cancer patients, the likelihood of developing lymphedema is approximately five times greater following postoperative RT compared to undergoing axillary lymph node dissection alone [17]. A comprehensive understanding of the mechanism(s) leading to a higher incidence of lymphedema following RT remains unclear.

RT inhibits lymphatic proliferation and survival by blocking the formation of compensatory lymphatic vessels, resulting from the downregulation of vascular endothelial growth factor receptor 3 (VEGFR-3) in lymphatic endothelial cells (LECs) in vitro and in vivo [18, 19]. On the other hand, one study has indicated that VEGF-C enhances the radiosensitivity of LECs by increasing their entry into the cell cycle, resulting in increased DNA damage, cell cycle arrest, and impaired lymphatic vessel repair [20]. Even though radiation can induce tissue swelling and inflammation, it often needs to be combined with the removal of lymph nodes to contribute to lymphedema progression in cancer patients [21]. The impact of RT on lymphatic growth, cell survival, and proliferation, and how these effects influence the maintenance of lymphatic function, remains unclear.

Radiation, while accomplishing tumor cell killing, also induces senescence in both tumor and normal tissues through the induction of DNA damage [22, 23]. Cancer cells may also reenter the cell cycle subsequent to a prolonged senescence arrest to produce progeny with resistance to therapy, chromosomal instability, or a cancer stem cell-like phenotype, providing a survival advantage [24, 25]. Therefore, senescent cells in tumors can, paradoxically, promote tumor relapse, metastasis, and resistance to therapy, in part, through the release of inflammatory factors, including the expression of the senescence-associated secretory phenotype (SASP) [25, 26]. In contrast, normal cells undergo senescence during aging, leading to a permanent blockage in cell proliferation and the release of SASP, contributing to chronic inflammation [27–30]. During lymphedema, activation of T cells and macrophages results in an inflammatory response leading to tissue remodeling, oxidative stress, and fibrosis [31]. Nevertheless, the contribution of radiation to senescence induction in LECs and its role in lymphedema are not well understood.

In this study, we found that radiation prevents LEC cell growth, accompanied by increased cell death and senescence in LECs. RT alone induces tail swelling and senescence in a mouse model, but upon lymphatic ablation in the tail, swelling and senescent cells were increased. Treatment with a senolytic agent reduced radiation-induced senescence while increasing cell death in LECs and in the tail lymphedema mouse model. This suggests that RT induces senescence, contributing to CRL, and that targeting senescent cells may represent a viable therapeutic strategy.

## Results

### Radiation prevents lymphatic endothelial cell growth and proliferation

**Fig. 1:**
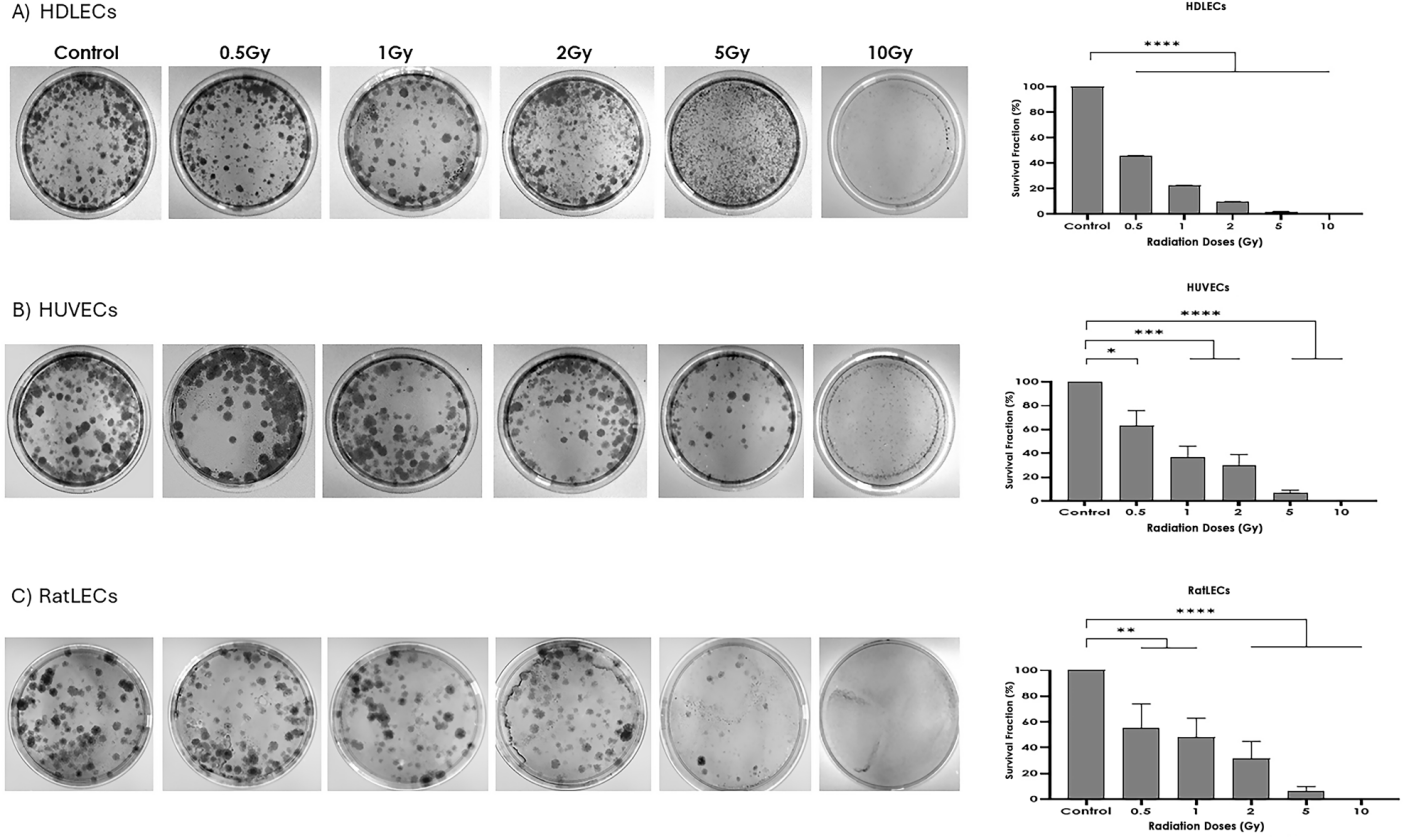
Radiation reduces colony-forming ability in endothelial cells. **A** HDLECs, **B** HUVECs, and **C** RatLECs were treated with 0.5, 1, 2, 5, and 10 Gy of radiation. Cells were then kept in the humidified incubator for 14 days (HDLECs), 10 days (HUVECs), and 7 days (ratLECs). Colonies were then fixed with methanol, stained with 0.5% crystal violet, and manually counted. Bar graphs represent survival fractions of colonies after RT in three independent experiments. Error bars represent mean ± SEM, *P < 0.05, **P < 0.01, ***P < 0.001, and ****P < 0.0001.

**Fig. 2:**
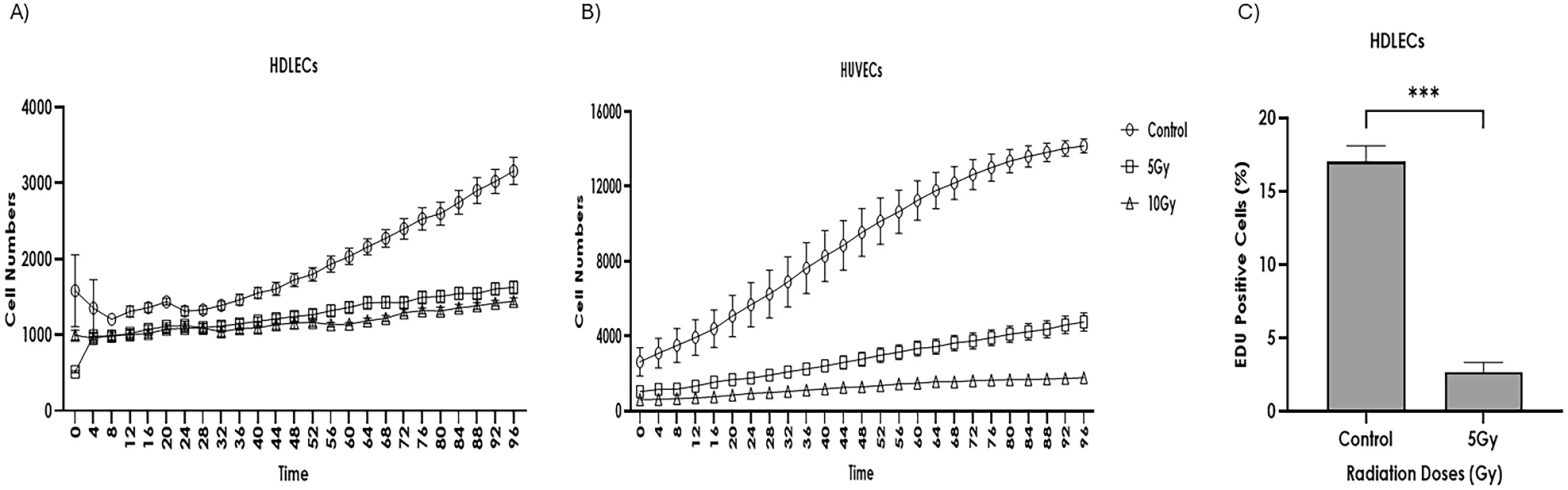
Radiation reduces cell numbers and proliferation in lymphatic endothelial cells. **A** HDLECs and **B** HUVECs were seeded in 96-well plates and treated with 5 and 10 Gy of RT, and the grown cells were imaged by a Cytation C10 every four hours for 96 h to monitor their response to treatment. **C** The bar graph represents the percentage of proliferating HDLECs after 48 h treatment with 5 Gy of radiation. Experiments represent three independent repeats. Error bars represented mean ± SEM, ***P < 0.001.

**Fig. 3:**
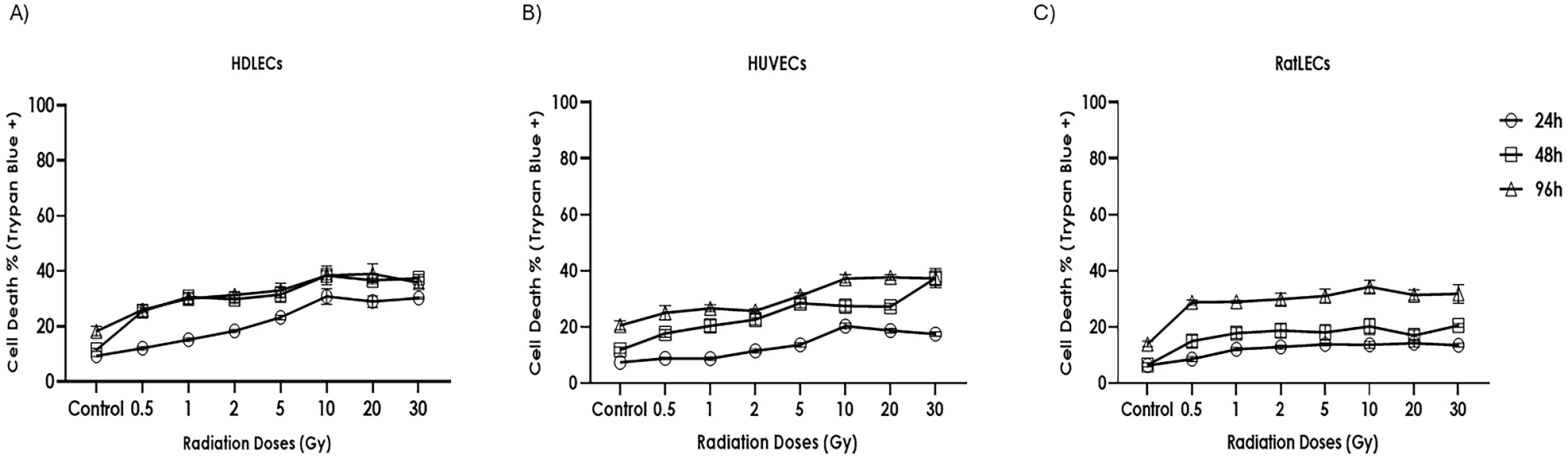
Radiation induces cell death in a dose-independent manner. **A** HDLECs, **B** HUVECs, and **C** ratLECs were exposed to a range of radiation doses (0.5–30 Gy) over a 96-h. time course. Cell death was determined by the trypan blue exclusion assay. Graphs represent the percentage of cell death after treatment in three independent experiments. Error bars represent mean ± SEM.

To determine whether radiation alters cell growth in LECs, a clonogenicity assay was conducted following exposure to a range of doses (0–10 Gy). RT dose as low as 0.5 Gy reduced the survival fraction to 50% in human dermal lymphatic endothelial cells (HDLECs), human umbilical vein endothelial cells (HUVECs), and rat mesenteric lymphatic endothelial cells (ratLECs) (Fig. 1). Similarly, RT significantly reduced cell numbers in both HUVECs and HDLECs over 96 h. In untreated cells, the cell number increased from over 3000 cells after 96 h, whereas in RT treated cells, the cell number remained at approximately 1000 cells after 10 Gy of radiation (Fig. 2A, B). To determine whether LEC proliferation was affected, an EDU proliferation assay was performed. This showed decreased EDU-positive HDLECs from 17% to 3% in the RT-treated group compared to the control, indicating reduced cellular proliferation (Fig. 2C). The reduced LEC growth could also be due to increased cell death. We found that RT increased cell death in HDLECs, HUVECs, and ratLECs over a range of doses (0.5–30 Gy) but plateaued at 35% cell death after 1 Gy RT. Furthermore, the plateau effect was observed even 96 h after RT treatment (Fig. 3A–C). To determine whether RT induced cell death via apoptosis, an annexin-V/7AAD assay was performed. This revealed that RT induced a plateau of 15% apoptotic cell death in HDLECs at 10 Gy after 48 h, similar to the total amount of cell death (Supplementary Fig. 1). To determine whether LECs were under cell cycle arrest (quiescence), cells were pretreated with growth factor VEGF-C, known to induce LEC proliferation [32, 33]. VEGF-C increased colony numbers in LECs, both alone and with low-dose RT (1 Gy), but not in higher RT doses (Supplementary Fig. 2A–C). Moreover, VEGF-C failed to decrease RT-induced cell death (Supplementary Fig. 2D–F), indicating that the cells were not in a quiescent state. Consistent with this, RT reduced VEGFR3 expression after 5 Gy treatment in HDLECs (Supplementary Fig. 2G–I). Taken together, these data indicate RT blocks LEC growth and proliferation.

### Radiation induces senescence in lymphatic endothelial cells

**Fig. 4:**
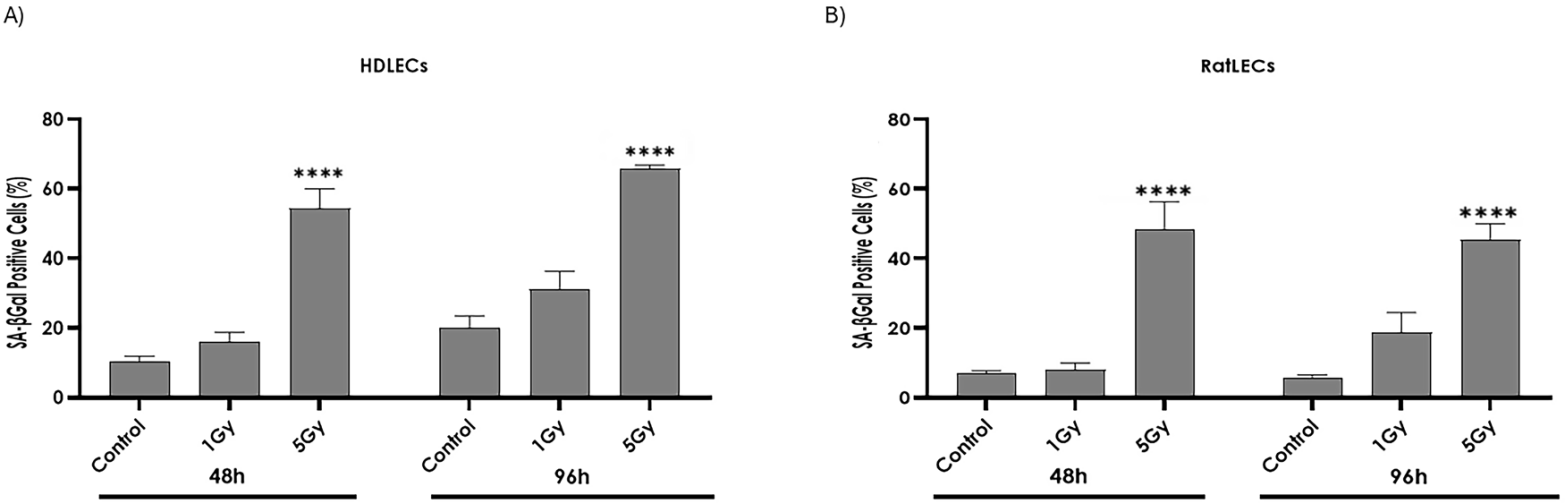
Radiation increases SA-βgal-positive cells in lymphatic endothelial cells. **A** HDLECs and **B** ratLECs were treated with 1 Gy and 5 Gy radiation for 48 h or 96 h. Senescent cells were detected by a SA-βgal assay. Bar graphs represent the percentages of SA-βgal-positive cells across six independent experiments. Error bars represent mean ± SEM, ****P < 0.0001.

**Fig. 5:**
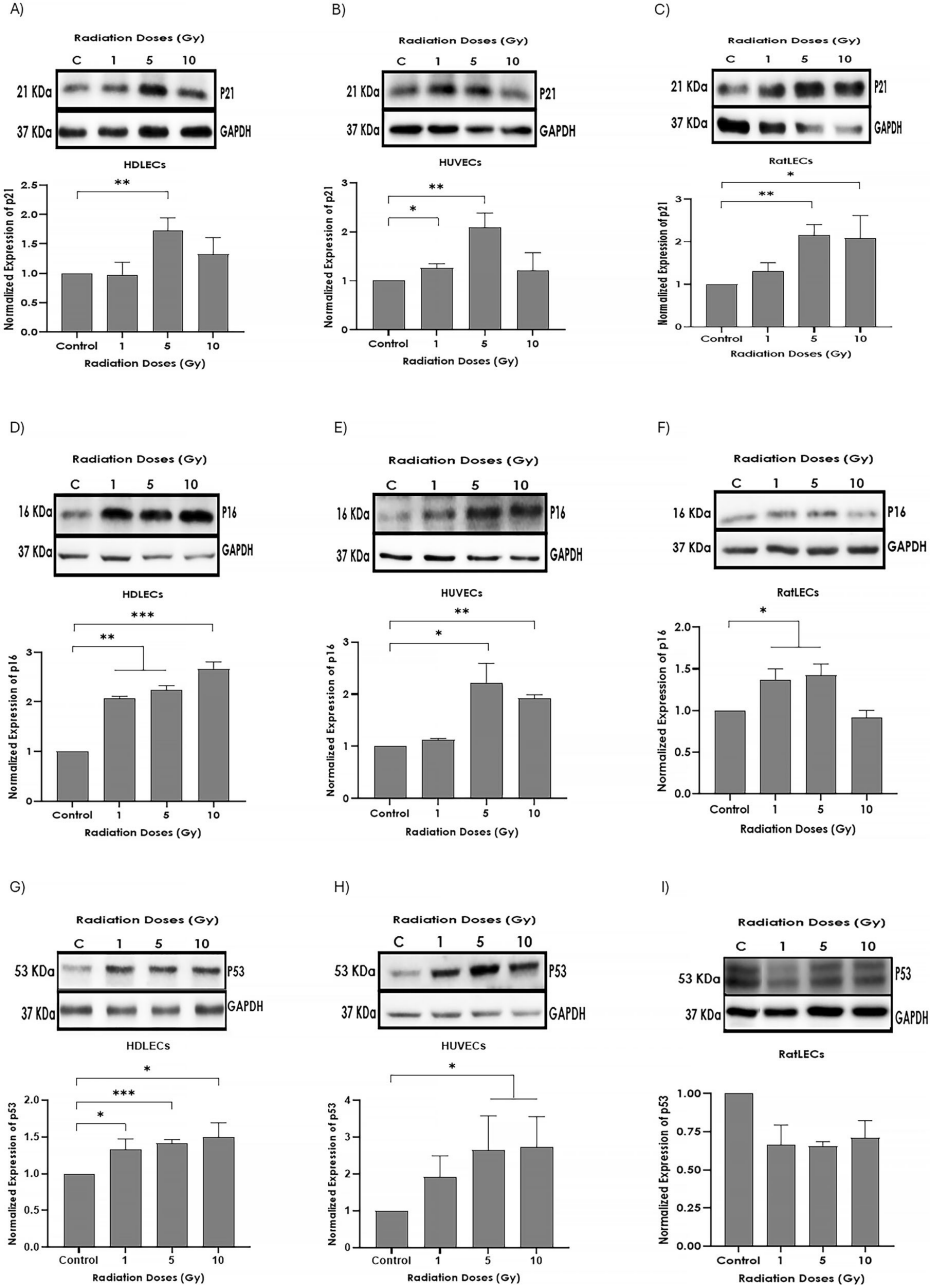
Radiation increases protein expression of senescence markers p21, p16, and p53 in endothelial cells. HDLECs, HUVECs, and ratLECs were treated with radiation (1, 5, and 10 Gy) for 48 h. The cells were lysed and western blotted for senescence markers p21 (**A**–**C**), p16 (**D**–**F**), and p53 (**G**–**I**). GAPDH protein levels were used as a loading control. Representative western blot images from three independent experiments are shown. Quantitative analysis is presented as bar graphs of protein expression levels normalized to the loading control. The error bars represent mean ± SEM, *P < 0.05, **P < 0.01, and ***P < 0.001.

Since lymphatic cell growth and proliferation were blocked following RT, and cell death did not increase proportionally with RT dose, we determined whether LECs are under permanent cell cycle arrest (senescence). Using a Senescence Associated Beta-Galactosidase (SA-βgal) assay, we showed a marked increase in SA-βgal positive cells following RT (5 Gy) in HDLECs (approx. 60%) and ratLECs (approx. 50%) at both 48 h and 96 h (Fig. 4 and Supplementary Fig. 3). The levels of SA-βgal positive cells remained unchanged after 96 h following RT treatment, suggesting that LECs had entered a stable senescent state rather than progressing to cell death. To further confirm whether LECs were in senescence, we determined the levels of other senescence markers, including p21, p16, and p53. We found that protein expression of p21 (Fig. 5A–C), p16 (Fig. 5D–F), and p53 (Fig. 5G–I) was increased in HDLECs, HUVECs, and ratLECs following a dose range of RT (1–10 Gy). Together, these findings demonstrate that RT induces senescence in LECs.

### Tail lymphedema is prolonged by radiation associated with increased cellular senescence and apoptosis

**Fig. 6:**
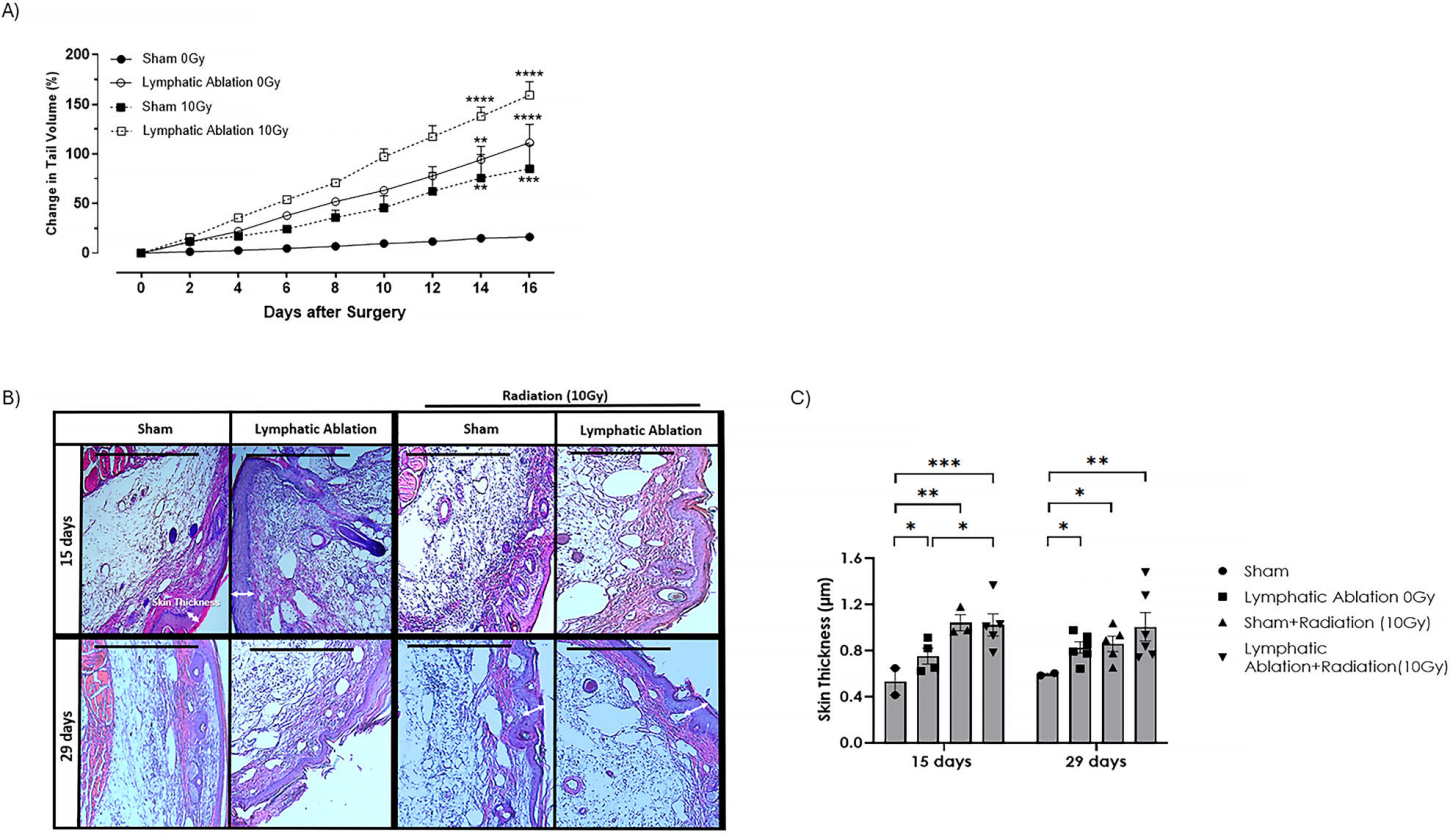
Radiation induces prolonged tail swelling in the lymphedema mouse model. Lymphedema was induced via lymphatic ablation in the tails of female C57BL/6J mice (6 weeks old), followed by 10 Gy radiation. Controls were sham surgery, which only involved skin incision, and mice without radiation treatment. **A** Tail volume measurements were obtained over 16 days. **B** Representative H&E staining from cross-sections of tail tissues at 15 days and 29 days after RT is presented to indicate skin thickness in tails compared to sham and lymphatic ablation alone. The double-sided white arrow shows skin thickness. Scale bar: 20 μm. **C** Quantification of skin thickness 15 days and 29 days post-RT was determined. Experiments represent five independent experiments, and error bars indicate mean ± SEM, *P < 0.05, **P < 0.01, ***P < 0.001, and ****P < 0.0001.

**Fig. 7:**
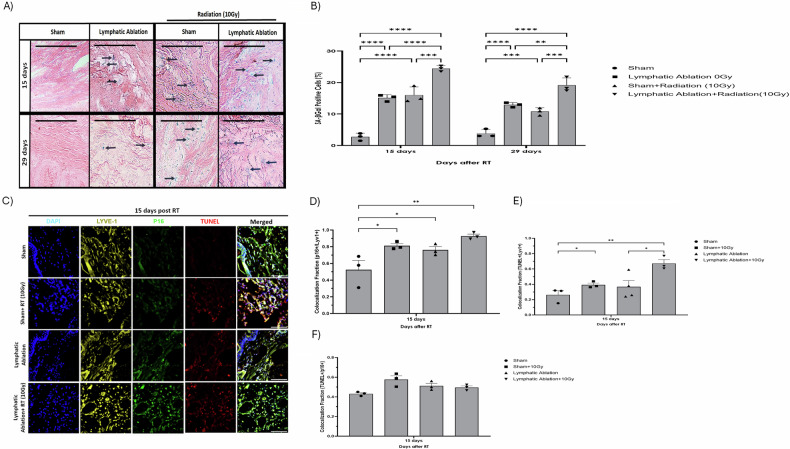
Radiation induces senescence in the murine tail lymphedema model. **A** Representative SA-βgal staining from cross-sections of tail tissues indicates higher levels of SA-βgal positive cells in sham+10 Gy, lymphatic ablation, and lymphatic ablation+10 Gy compared to sham control in both 15- and 29-days post treatment. Arrows point to SA-βgal-positive cells. Scale bar: 20 μm. **B** Percentages of SA-βgal positive cells 15 days and 29 days post-RT. **C** Representative immunofluorescence staining of LYVE-1-positive lymphatic endothelial cells (yellow), senescence marker p16 (green), and apoptosis (TUNEL) (red) in tail tissues from lymphedema mice 15 days post-RT. Bar graphs represent the percentage of cells with (**D**) p16+ LYVE-1+ colocalization fraction, **E** TUNEL + LYVE-1+ colocalization fraction, and **F**) p16+ TUNEL+ colocalization fraction. Experiments represent three independent repeats, and error bars represent mean ± SEM, *P < 0.05, **P < 0.01, ***P < 0.001, and ****P < 0.0001. Scale bar: 100 μm. LYVE-1 lymphatic vessel endothelial hyaluronan receptor-1, TUNEL terminal deoxynucleotidyl transferase dUTP nick end labeling, DAPI 4′, 6-diamidino-2-phenylindole.

Since RT induces senescence in LECs, we next evaluated its effects on swelling and cellular senescence in a murine tail lymphedema model. In sham-operated mice, 10 Gy localized radiation increased tail swelling compared to the untreated sham controls. However, in the lymphatic-ablated tails, radiation further increased swelling compared to the untreated lymphedema tail after 15 days (Fig. 6A). By 29 days post-RT, the tail swelling was starting to resolve (Supplementary Fig. 4). In Hematoxylin and Eosin (H&E)-stained tail sections, sham tails treated with radiation, and lymphatic ablated tails treated with radiation showed a significant increase in subcutaneous tissue thickness compared to the sham control, 15- and 29-days post RT (Supplementary Fig. 5). Lymphatic ablation only and radiation (10 Gy) only significantly increased skin thickness compared to sham controls at 15 and 29 days, while the combination of lymphatic ablation and radiation resulted in the strongest effect across time points (Fig. 6B, C). To assess senescence in vivo, tail tissues were stained for SA-βgal at 15 days and 29 days post-RT. The number of SA-βgal positive cells increased in sham with radiation (10 Gy) tails, and lymphatic ablated tails (17%) compared to sham controls (4%), however, the SA-βgal positive cells in the lymphatic ablated tails treated with radiation (10 Gy) increased further to 25% 15 days post-treatment (Fig. 7A, B). To further confirm the presence of senescence in LECs, the tail sections were co-stained for a lymphatic cell marker, LYVE1, and a senescence marker, p16. RT treated sham and/or lymphatic ablated tails increased p16 expression in LYVE1-positive cells compared to sham tails 15- and 29-days post RT (Fig. 7C, D and Supplementary Fig. 6A, B), supporting the induction of lymphatic senescence by RT in vivo. Since we found RT also induces cell death in LECs, we determined the amount of cell death using TUNEL staining.

We found that sham and lymphatic ablated tail tissue following RT treatment had an increased number of LYVE-1 and TUNEL positive cells, indicating increased RT induced cell death in lymphatic cells 15 days post treatment (Fig. 7C, E). In contrast, p16-positive lymphatic cells were negative for TUNEL staining (Fig. 7C, F). Taken together, this indicates that lymphatic senescence cells are present in lymphedema tissue and are resistant to radiation-induced cell death.

### Radiation therapy induces a senescence-associated phenotype in cancer-related lymphedema patients

**Fig. 8:**
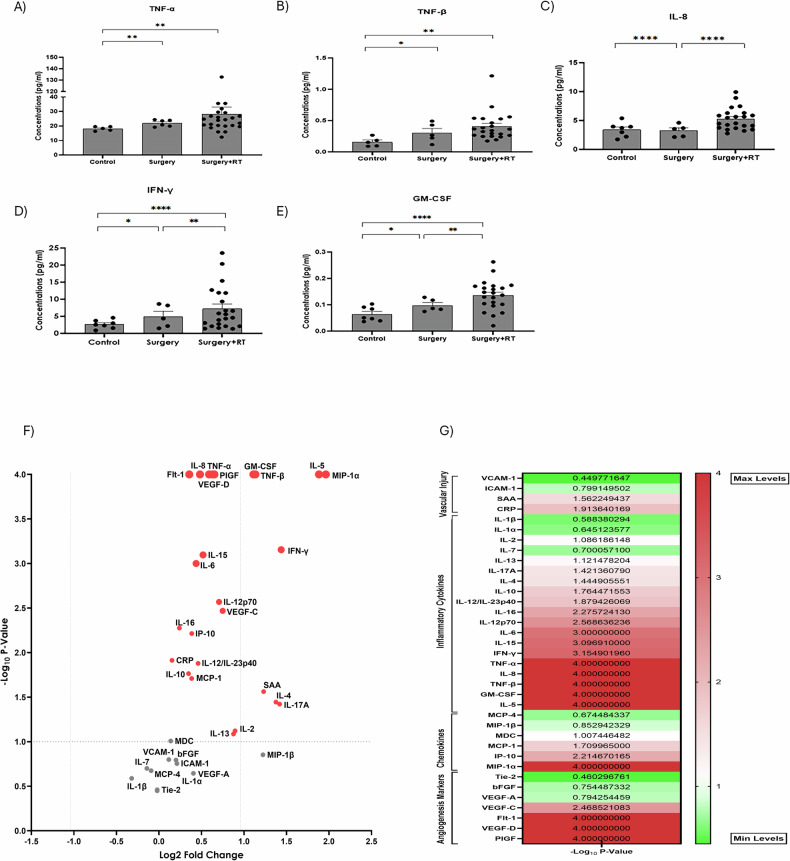
Inflammatory factors associated with the SASP in patient plasma samples. The fold changes in 39 cytokines, chemokines, and growth factors were determined in plasma samples from cancer-related lymphedema patients compared with non-lymphedema controls (Table 1) using the MesoScale Discovery Cytokine Kit. Bar graphs represent expression levels of inflammatory cytokines: **A** TNF-α, **B** TNF-β, **C** IL-8, **D** IFN-γ, **E** GM-CSF in plasma samples of lymphedema patients who underwent surgery, and surgery+RT compared to normal controls. The dots indicate the number of individuals in each group. The fold changes were depicted by (**F**) volcano plot and **G** heat map representing differential levels of vascular injury factors, inflammatory cytokines, chemokines, and angiogenic and lymphangiogenic markers. Experiments represent three independent repeats, and error bars represent mean ± SEM, *P < 0.05, **P < 0.01, and ****P < 0.0001.

**Fig. 9:**
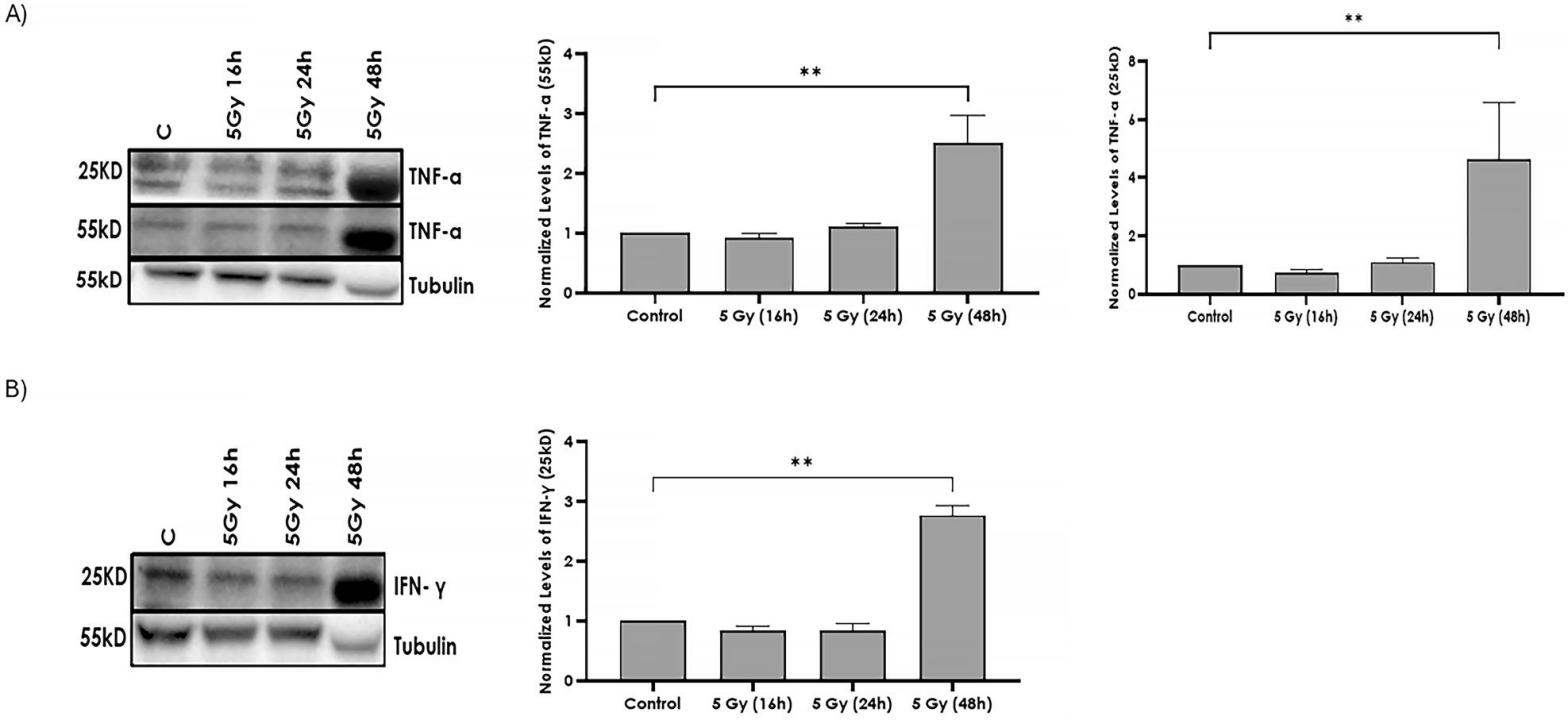
TNF-α and IFN-γ were upregulated in HDLECs following RT. Protein expression levels of (**A**) TNF-α and **B** IFN-γ after radiation treatment (5 Gy) in lysates of HDLECs were determined in 48 h by western blotting. Tubulin was used as a loading control. Densitometry of expression levels of TNF- α and IFN- γ were normalized to Tubulin from three independent experiments. Error bars represent mean ± SEM, **P < 0.01.

Senescent cells are known to secrete various immune and angiogenic factors as a part of the Senescence Associated Secretory Phenotype (SASP), exacerbating inflammation [25, 26, 34]. We evaluated 33 cancer patients (mostly females) with secondary lymphedema post-treatment (surgery, and/or radiotherapy) and 7 non-lymphedema controls. Most CRL patients (91%) were aged 32–88 years, with 70% having breast CRL and 30% linked to other cancers (Table 1). Plasma was isolated from lymphedema patients’ blood samples, and cytokine and chemokine levels were determined using a MesoScale assay. The inflammatory cytokines interleukin-10 (IL-10), IL-12p70, IFN-γ, tumor necrosis factor-alpha (TNF-α), TNF-β, IL-8, and granulocyte-macrophage colony-stimulating factor (GM-CSF) levels were higher in RT -treated CRL patients’ plasma compared to CRL patients with surgery alone or non-lymphedema controls (Fig. 8A–E and Supplementary Fig. 7). However, the levels of cytokines were not affected by the amount of time elapsed from receiving RT (Supplementary Fig. 8). Moreover, the levels of Monocyte Chemoattractant Protein-1/ C-C motif chemokine ligand 2 (MCP-1/CCL-2), Macrophage Inflammatory Protein-1α (MIP-1α/CCL-3), and interferon-γ–inducible protein-10 (IP-10) were higher in radiation-treated patients’ plasma samples compared to non-lymphedema controls (Fig. 8F, G). To confirm that lymphatic cells secrete these factors, HDLECs were treated with 5 Gy of radiation over a 48- and 72-h time course, and the supernatants were used to evaluate 36 different cytokines and chemokines, and the lysates were also western blotted for inflammatory cytokines TNF-α and IFN-γ. We found that a number of cytokines and chemokines including MCP-1/CCL-2, CCL-5/RANTES (Regulated upon Activation, Normal T-cell Expressed and Secreted), C-X-C motif chemokine ligand 1 (CXCL-1/ Growth-Related Oncogene (GROα)), CXCL-12, G-CSF, GM-CSF, IL-8, Macrophage Inflammatory Protein (MIF), and Serpin E1/ Plasminogen Activator Inhibitor-1 (PAI-1) were higher in RT -treated HDLECs compared to untreated cells over 48 and 72 h (Supplementary Fig. 9 and Supplementary Table 1). Both TNF-α and IFN-γ protein levels increased after 48 h of RT (Fig. 9), correlating with a SASP response and consistent with lymphedema patients’ plasma samples.

**Table 1:** Lymphedema patients’ characteristics.

	Value	Range
Non-lymphedema controls		
Sample size (*n*)	7	-
Female (*n*)	4	-
Male (*n*)	3	-
Age (years)	43 ± 6.27^a^	32–63
BMI (kg/m^2^)	26.7 ± 2.27^a^	19.9–38.6
Patients with secondary lymphedema		
Sample size (*n*)	33	-
Female (*n*)	30	-
Male (*n*)	3	-
Age (years)	65 ± 2.12^a^	32–88
BMI (kg/m^2^)	28.55 ± 1.09^a^	21.16–57.53
Breast cancer-related lymphedema	23	-
Other cancers-related lymphedema	11	-
Surgery treatment	32	-
Surgery+radiation treatment (<10 years)	17	-
Surgery+radiation treatment (>10 years)	6	-

Plasma samples from 33 patients (mostly females) with secondary lymphedema following cancer treatment, and as a comparison, 7 non-lymphedema controls were chosen for cytokine analysis with the MesoScale Cytokine Kit. Most of these patients (91%) ranged in age from 32 to 88 years. Approximately 70% had a history of breast CRL, 30% developed lymphedema due to other types of cancer.

^a^Represent mean ± SEM.

### Senolytic agents induce cell death in radiation-induced senescent LECs

**Fig. 10:**
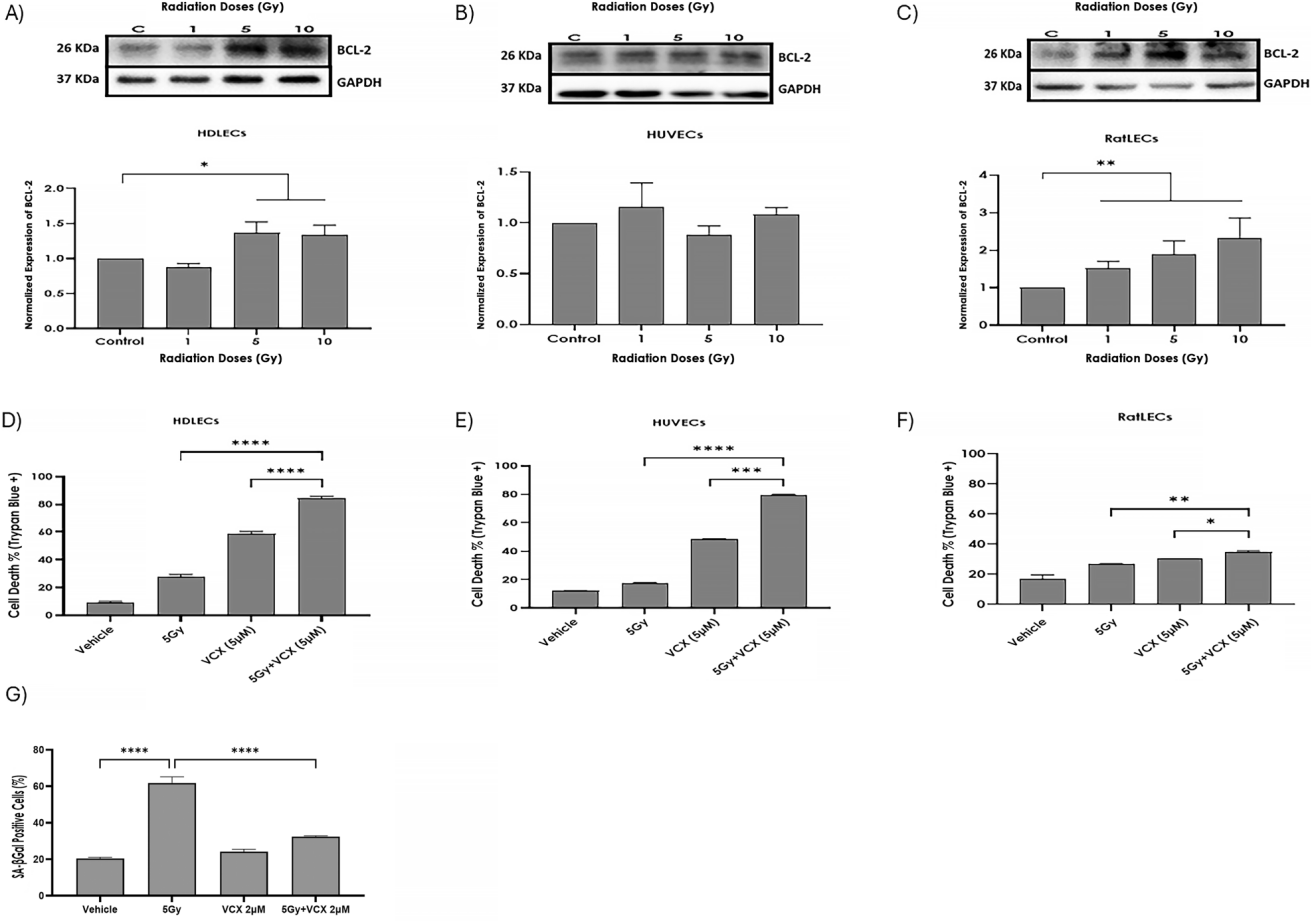
Senolytic agent venetoclax (VCX) induces cell death in senescent RT-treated cells. **A** HDLECs, **B** HUVECs, and **C** ratLECs were treated with 1, 5, and 10 Gy radiation for 48 h, and the cells were then lysed. Expression levels of BCL-2 were determined by western blot, and the loading control was GAPDH. Densitometry of BCL-2 expression was normalized to GAPDH. The amount of cell death was determined by trypan blue exclusion assay in was performed in (**D**) HDLECs, **E** HUVECs, and **F** ratLECs after radiation treatment in the presence or absence of VCX (5 μM). **G** The percentages of SA-βgal positive HDLECs after treatment with 5 Gy of radiation in the presence or absence of VCX (2 μM) were determined. This represents three independent experiments, and error bars represent mean ± SEM. *P < 0.05, **P < 0.01, ***P < 0.001, and ****P < 0.0001.

In tumors, senolytic agents have been demonstrated to selectively kill senescent cancer cells and reduce SASP factors [35–37]. Venetoclax (VCX) (ABT-199) is a senolytic agent that inhibits the anti-apoptotic protein BCL-2 and is used for the treatment of multiple cancers [36, 38]. We found that 5 and 10 Gy RT increased BCL-2 protein levels in HDLECs and ratLECs, but not in HUVECs, as determined by Western blotting (Fig. 10A–C) and confirmed by immunostaining in HDLECs (Supplementary Fig. 10). In addition, BCL-2 expression co-localized to the mitochondria after RT (Supplementary Fig. 10). This correlated with HDLECs showing high BCL-2 expression with high p16 levels (senescence marker) and lower TUNEL staining (cell death) (Supplementary Figs. 11 and 12). This indicates BCL-2 is protecting HDLECs from RT-induced cell death. Co-treatment of these cells with 5 Gy radiation and VCX (5 µM) induced 80% cell death, whereas each treatment alone induced 55% and 25%, respectively, in both HDLECs and HUVECs (Fig. 10D, E). RatLECs treated with radiation and VCX showed only 30% cell death (Fig. 10F). To confirm that the increase in cell death is selective to senescent cells, we performed a SA-βgal assay. VCX (2 μM) reduced SA-βgal positive cells from 60% to 30% (Fig. 10G) and reduced BCL-2 and p16 protein expression levels in RT-treated HDLECs (Supplementary Fig. 13). We also found that using a combination treatment of another two senolytic agents, quercetin (174070100, Thermo Fisher Scientific) (Q, 25 µM), and dasatinib (SML2589, Sigma-Aldrich) (D, 25 µM) [39]. increased cell death in HDLECs after 5 Gy RT (Supplementary Fig. 14). This suggests that senolytic agents selectively induce cell death in RT induced senescent LECs.

**Fig. 11:**
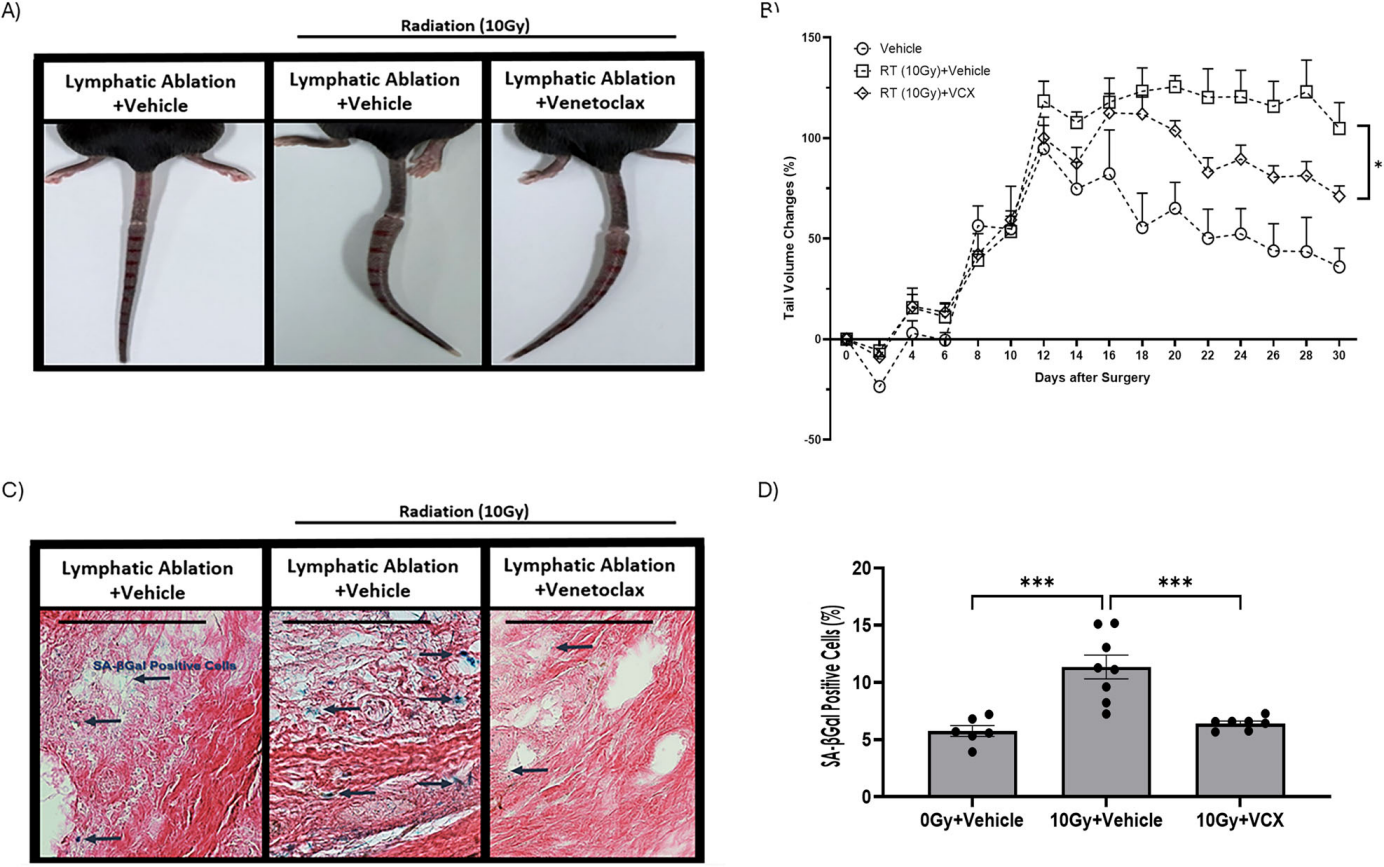
The effect of VCX on tail swelling in the radiation-induced tail lymphedema model. **A** Representative image of mouse tails following lymphatic ablation under different treatment conditions: vehicle control (0.5% CMC), radiation (10 Gy), and radiation (10 Gy) combined with VCX (100 mg/kg body weight of mouse in a concentration of 20 mg/ml). **B** Quantification of tail volume changes over 30 days post-surgery. **C** Representative images of SA-βgal staining in mouse tail tissues following lymphatic ablation with radiation (10 Gy) under different treatment conditions: vehicle and VCX. Arrows indicate SA-βgal positive cells. **D** Quantification of SA-βgal positive cells (%) across experimental groups. Experiments represent 6–8 independent repeats, and error bars represent mean ± SEM. *P < 0.05 and ***P < 0.001. Scale bar: 20 μm.

To determine whether RT increased BCL-2 expression affects tail lymphedema, after lymphatic ablation surgery and RT (10 Gy), mice were treated with VCX (100 mg/kg, formulated at 20 mg/ml) or vehicle for two weeks. VCX treatment reduced tail swelling, skin thickness, and collagen deposition by approximately 50% at 30 days compared with RT-treated mice receiving vehicle (Fig. 11A, B and Supplementary Figs. 15 and 16). After 30 days, the lymphedema tissue also showed reduced senescence markers (SA-βgal and p16 staining) and reduced immune cell infiltration (macrophages and CD4 + T cells) following VCX treatment (Fig. 11C, D and Supplementary Figs. 17–19). To confirm that VCX was targeting BCL-2 expressing cells, we found that BCL-2 expression was reduced (Supplementary Fig. 17) and cell death (TUNEL staining) was increased in the VCX-treated tails compared to RT and surgery alone (Supplementary Fig. 20). This suggests that the senolytic agent VCX can reduce RT induced tail lymphedema by eliminating lymphatic senescent cells.

## Discussion

As a therapeutic modality in cancer, RT increases overall survival, disease-free survival, and decreases regional recurrence [40, 41]. Unfortunately, RT is a significant risk factor for the development of CRL [3, 9]. Some clinical studies have indicated that combining RT and surgery increases the incidence and severity of breast CRL [42]. We have shown that RT induces senescence and cell death in lymphatic cells and in the murine tail lymphedema model, where RT increases swelling and senescence. In lymphedema patients who received RT, we found increased inflammation factors associated with SASP. Furthermore, we found upregulation of the anti-apoptotic protein BCL-2 after RT in LECs, and that using a BCL-2 inhibitor, VCX, in combination with radiation, selectively induced cell death in senescent LECs. Taken together, this indicates that RT induced senescence plays an important role in CRL progression.

Lymphatic proliferation is blocked by RT by inhibiting the formation of compensatory lymphatic vessels [18, 19]. One of the key regulatory factors in lymphangiogenesis, a process of producing lymphatic vessels from pre-existing vessels, is VEGF-C [32, 43]. VEGF-C binds to VEGFR-3, which is primarily found on LECs in normal adults and activates signaling pathways that lead to cellular proliferation, invasion, and migration [33, 44]. In addition, downregulation of VEGFR3 expression following radiation contributes to decreased LEC and lymphatic vessel numbers since activation of VEGFR-3 is required for LEC survival and proliferation [19]. This is consistent with our in vitro findings that VEGFR3 expression is reduced after RT. The induction of senescence may further impair lymphatic repair by limiting proliferative capacity and reducing responsiveness to lymphangiogenic signals, as supported by decreased VEGFR3 expression and lack of rescue by VEGF-C.

Exposure to RT causes several physiological changes that can lead to DNA double-strand breaks (DSBs), thereby initiating genomic instability and metabolic system dysfunction [22, 23, 40]. Activation of the DNA damage response (DDR) system triggers a cascade of different cellular pathways [45, 46]. DDR controls the cell cycle through increased expression of cell cycle arrest proteins p21 and p16, and activation of the tumor suppressor p53 [45]. If damage fails to be repaired, the cell either dies by apoptosis or enters senescence [47, 48]. In RT treated LECs, we found that cell cycle arrest proteins p21 and p16, and tumor suppressor p53 protein levels are increased, as well as senescence and cell death occurred. This suggests that DDR leads to senescent LECs and will be the focus of future investigation.

Although radiation effectively eradicates tumor cells, it also induces senescence in both malignant and healthy tissues by causing DNA damage [22, 23]. However, certain cancer cells may evade extended senescence and resume proliferation, leading to the emergence of progeny that exhibit therapeutic resistance, chromosomal instability, or characteristics resembling cancer stem cells; factors that collectively contribute to enhanced cellular survival [24, 25]. Consequently, senescent tumor cells may paradoxically promote tumor recurrence, metastasis, and resistance to therapy, in part by releasing pro-inflammatory factors characteristic of SASP [25, 26]. In normal cells, RT delivered either pre- or postoperatively induces senescence, resulting in irreversible growth arrest and the secretion of SASP factors that drive chronic inflammation [27–30]. Our findings indicate a similar effect of RT on LECs.

In lymphedema, there is an inflammatory response driven by macrophages and T cells, similar to the tumor microenvironment [7, 31, 49]. Indeed, chronic inflammation is a key feature of lymphedema, where macrophages and T cells infiltrate lymphedema tissue and increase inflammatory factors and lymphangiogenic growth factors, including VEGF-C [49–51]. Eliminating T cells significantly reduces lymphedema in mouse models, and blocking inflammatory factors IL-13 and IL-4 reduces lymphedema [52, 53]. In this study, we found increased inflammatory factors in lymphedema patients following RT associated with SASPs and further determined that these inflammatory factors remain elevated 10 years after RT. This prolonged inflammation over time can contribute to excess adiposity, extracellular matrix deposition, and the formation of dense fibrous tissue, including a population of senescent cells [31]. Using pharmacological targets to reduce inflammation in lymphedema has been investigated.

Ketoprofen, a well-known non-steroidal anti-inflammatory drug, in an open-label study, improved lymphedema skin pathology but failed to improve limb swelling [54]. In addition, 5-lipoxygenase metabolite leukotriene B4 (LTB4) has been shown to be an important mediator of lymphedema inflammation, and using a selective LTB4 antagonist, bestatin (Ubenimex), has been shown to reduce lymphedema and is being investigated in a multicenter placebo-controlled trial (NCT02700529) [55]. This illustrates the importance of inflammation in lymphedema.

The accumulation of senescent cells in different organs is a precursor of fibrosis development, neurological disorders, or chronic diseases, which have spurred interest in developing targeted therapies for senescent cells [56]. In chronic respiratory inflammation, senescence plays an important role in lung fibrosis, where the elimination of senescent fibroblasts reduces fibrosis [57]. Fibrous tissue in lymphedema is often present and leads to infection and pain [8]. We observed that radiation increased lymphatic vessel dilation, subcutaneous and cutaneous tissue thickness, fibrosis, and increased senescence in the mouse tail. Endothelial cells are relatively resistant to radiation-induced apoptosis, but they readily undergo senescence or permanent cell-cycle arrest after exposure to a moderate or high radiation dose [58, 59]. Our results demonstrated that LECs and mouse tails treated with radiation (with or without lymphatic ablation) enter a senescent state, exhibiting intrinsic resistance to apoptotic stimuli, particularly with higher doses of radiation. Moreover, RT-induced senescent cells and RT-treated tail lymphedema tissue had higher expression levels of BCL-2. The selective upregulation of BCL-2 in RT-induced senescent LECs identifies a targetable vulnerability, as these cells rely on pro-survival signaling to persist. Thus, using senolytic drugs that target senescent cells by inhibiting the pro-survival pathways could be efficacious [35]. Indeed, VCX (a BCL-2 inhibitor) treatment reduced swelling and senescence markers while increasing apoptosis in mice with RT treated tail lymphedema, consistent with selective clearance of senescent cells. This supports a model in which removal of senescent LECs alleviates inflammation and tissue remodeling, leading to improved lymphatic function. VCX or the combination of quercetin (Q), a plant-derived flavonoid that targets PI3K/AKT pathway modules, and dasatinib (D), a small-molecule tyrosine kinase inhibitor, has been shown to eliminate senescent cells by inducing apoptosis, consistent with our study [60, 61]. However, VCX induces toxicities such as bone marrow suppression and edema in acute myeloid leukemia patients, suggesting effects on non-senescent cells [62–64].

In conclusion, our study identifies RT as a potent inhibitor of LEC growth, mediated by both senescence and cell death in vitro and in vivo. In CRL patients and LECs treated with RT, we observed increased levels of SASP cytokines and chemokines. Importantly, treatment with senolytic agents induced cell death in senescent LECs. Taken together, these findings indicate that RT-induced LEC senescence contributes to lymphedema and might represent a promising therapeutic target to treat CRL in cancer survivors. In the future, the effect of other senolytic agents on lymphedema swelling, SASP, and inflammation will be investigated to potentially be translated to lymphedema treatments.

## Materials and methods

### Blood sample collections and SASP measurement

To assess changes in inflammatory and senescence-associated cytokines and chemokines in CRL, we quantified 39 different biomarkers in plasma samples using the MSD Cytokine Kit (K15209G-1, MesoScale Discovery Inc.). Anticoagulated venous blood samples were processed at 1500 × *g* for 15 min at 4 °C and stored at −80 °C. Cytokine levels were normalized to control values and visualized using GraphPad Prism (v10.2.3) for heat map and volcano plot analysis.

### Cell culture and cell lines

Three distinct cell lines were employed in the study: human dermal lymphatic endothelial cells (HDLECs, C-12217) sourced from PromoCell in Heidelberg, Germany; human umbilical vein endothelial cells (HUVECs, C0035C) obtained from Thermo Fisher Scientific; and rat mesenteric lymphatic endothelial cells (ratLECs), generously provided by Dr. Pierre-Yves von der Weid at the University of Calgary in Canada. These cell lines were cultured on 100 mm Petri dishes (Sarstedt, Montreal, QC, Canada) coated with 0.2% gelatin (Sigma-Aldrich). They were maintained in Endothelial Cell Growth Medium-2 with supplements (C-22121, PromoCell) within a humidified incubator set to 37 °C with 5% CO_2_. For experimental purposes, cells were utilized between passages 5 and 8.

### Mice tail lymphedema model

A total of 41 female C57BL/6J mice (6 weeks old) were obtained from Charles River Laboratories and housed at the North Campus Animal Services (NCAS) facility at the University of Alberta. Mice were maintained in a controlled environment with a 12-h light-dark cycle and randomly assigned to four groups, with five mice assigned to each group. In the first group, acquired lymphedema was induced via lymphatic trunk ablation in the tail under anesthesia (2–3% isoflurane (DIN 02237518, Fresenius Kabi Global)) and maintained on a heating pad to keep their body temperature at 36.5–37.5 °C. A 3-mm circumferential incision was made, and lymphatic trunks were removed using microsurgical scissors. The second (sham) group underwent a skin incision without lymphatic ablation. The third group received local RT (10 Gy) one day post-ablation, while the fourth (sham+RT) group underwent tail skin excision followed by radiation without lymphatic ablation.

In a separate experimental cohort, all mice underwent lymphatic trunk ablation and were subsequently assigned to receive vehicle (0.5% carboxymethyl cellulose (CMC; 419303, Sigma-Aldrich)) (*n* = 6) alone, RT (10 Gy) with vehicle (*n* = 8), or RT combined with VCX (ABT-199; GDC-0199; RG7601, MedChem Express) (100 mg/kg, formulated at 20 mg/mL) (*n* = 7), administered by oral gavage. Mice exhibiting tail necrosis or infection were excluded, including one animal from the RT + VCX group that developed necrosis. Female mice were used to align with our patient cohort, in which most individuals with lymphedema were female. Tail circumference was measured every other day using a digital caliper, and tail volume was determined using the truncated cone equation. After 30 days, mice were euthanized, and cardiac blood samples were stored at −80 °C for analysis. Tail tissues were collected for paraffin-embedded and frozen section preparation.

### Radiation treatment (RT)

RT was performed using a MultiRad 160S/N 2329a60114. For in vitro experiments, settings were optimized on 85 kV and 15 mA, delivering radiation at 1.5 Gy/min with doses ranging from 0.5 to 30 Gy, using a standard 0.5 mm aluminum (Al) filter. For in vivo studies, a single dose of 10 Gy was delivered using a standard 0.3 mm copper (Cu) filter under the same voltage and current settings.

### Venetoclax (VCX) treatment

Forty-eight hours after radiation (5 Gy), cells were treated with VCX (2 or 5 µM) or vehicle control (DMSO, final concentration ≤0.1%) for 24 h, after which they were collected for cell viability assays, Senescence Associated Beta-Galactosidase (SA-βgal) staining, and Western blotting analysis. In the second mouse experimental cohort, two groups received local RT (10 Gy) one day after lymphatic ablation. Six days after, mice were treated by oral gavage with either vehicle (0.5% CMC) or VCX (100 mg/kg, formulated at 20 mg/mL). A third group, which did not receive RT, was treated with vehicle alone. Treatments were administered daily for 2 weeks, and body weight and tail volume were monitored every other day throughout the treatment period. Following completion of treatment, mice were maintained for an additional week before euthanasia.

### Cell viability assays

To investigate the effects of radiation on lymphatic cell death and survival, multiple viability assays were used after treating HDLECs and ratLECs with various doses of radiation (0.5–30 Gy). HUVECs served as a control for the vasculature system. Cell viability was assessed using the trypan blue exclusion assay, where cells were stained with 0.04% trypan blue (Sigma–Aldrich). Following treatment, both adherent and non-adherent cells were collected, resuspended in PBS, and stained at a 1:1 ratio. Viable cells remained unstained, whereas non-viable cells appeared blue. Cell viability was quantified using a hemocytometer, with total cell death calculated as (dead cells / total cells) × 100. To evaluate apoptosis and overall cell death, the annexin V/7-AAD assay was employed, where cells were stained with annexin V-FITC (51-65874X, BD Biosciences, San Jose, CA, USA) and 7-AAD (51-66211E, BD Biosciences, San Jose, CA, USA). Apoptotic cells were identified by annexin V positivity, and data acquisition was performed using the Attune NxT flow cytometer (A28997, Life Technology, Thermo Fisher Co). A total of 10,000 events were analyzed using FlowJo software (v10.10, Tree Star Inc).

For long-term cell survival assessment, a clonogenicity assay was conducted. Cells were seeded at a low density and cultured for 7–14 days after treatment to allow colony formation. If any stimulator or inhibitor (10 ng/ml VEGF-C) was tested, cells were pretreated with the stimulator or inhibitor for 1 h; then, the plates were treated by RT (0–10 Gy). Colonies were then fixed with methanol (34860-4L-R, Sigma), stained with 0.5% crystal violet (C6158, Sigma-Aldrich, St. Louis, MO, USA), and manually counted. A colony was defined as a cluster containing ≥50 cells. Given the varying adherence of cells, plating efficiency (PE) was measured, and based on that, survival fraction (SF) was calculated to assess cell survival. To confirm the rate of cell growth, the cell numbers were evaluated in the presence or absence of RT. Cells were seeded in 96-well plates, and grown cells were imaged by a Cytation C10 confocal imaging reader (BTC10MPWC, Agilent Technologies) every four hours for 96 hours to monitor their response to treatment. To evaluate cellular proliferation, an EdU (5-ethynyl-2’-deoxyuridine) proliferation assay was performed based on the EdU staining proliferation kit’s instructions (ab219801, Abcam), and proliferating cells were quantified using an Attune NxT flow cytometer (A28997, Life Technology, Thermo Fisher Co).

### Senescence associated beta-galactosidase (SA-βgal) Assay

To determine levels of SA-βgal positive cells, cells were treated with different doses of radiation (1 and 5 Gy). After the designated incubation period, cells were stained according to kit instructions (Senescence β-Galactosidase Staining Kit #9860 Cell Signaling). Blue-stained cells were examined under a light microscope (200X magnification) across 5–10 fields to quantify SA-βgal-positive cell percentages. To evaluate SA-βgal-positive cells in the tail tissue, frozen sections were prepared using Optimal Cutting Temperature (OCT) compound for cryosectioning. Ten-micrometer sections were air-dried at room temperature before SA-βgal-staining, following the protocol described above. For counterstaining, the slides were immersed in 0.25% eosin for 5 s to give the slides a light pink background. The slides were then mounted by Permount (VECTASHIELD® Antifade Mounting Medium, H-1000-10, Vector Laboratories) and covered with a glass coverslip. Brightfield imaging was performed using an Optika B-290TB (Optika, Ponteranica, Italy) microscope. Quantification of SA-βgal-positive areas was performed using Fiji software version 1.54 f (National Institutes of Health, Bethesda, MD, USA) with a minimum of five high-powered fields per animal.

### Hematoxylin and Eosin (H&E) Staining

To evaluate the subcutaneous and skin thickness in tails, tissues were fixed overnight in 10% formalin (23-245684, Fisher Scientific, Waltham, MA, USA), decalcified using 10% ethylenediaminetetraacetic acid (EDTA; E5134, Sigma-Aldrich, St. Louis, MO, USA), and subsequently embedded in paraffin. Hematoxylin (Modified Harris Hematoxylin, 22-050-206, Fisher Scientific) and Eosin (318906, Sigma) (H&E) staining were performed using 5 µm sections.

Slides were deparaffinized at 65°C for 10 min, followed by two xylene (208002, Fisher Chemical™) washes (5 min each) to remove residual paraffin. Rehydration was performed using a graded ethanol (P016EAAN, Greenfield Global) series (100% ethanol twice for 5 min, 90% ethanol for 5 min, 80% ethanol for 5 min, and 70% ethanol for 5 min). Slides were then washed in deionized water (10 min) and stained with hematoxylin (20 min), followed by rinsing in deionized water until clear.

For differentiation, slides underwent ten quick dips in 1% acid alcohol, rinsed in PBS (30 s), and washed in deionized water (3 min). If necessary, slides were re-stained in hematoxylin; otherwise, they were stained with eosin (30 s) and dehydrated in an ascending ethanol series (95% ethanol twice for 1 min, 100% ethanol twice for 1 min). Slides were then cleared in xylene twice (1 min each), mounted with Permount and a coverslip, and dried overnight at room temperature. H&E-stained slides were evaluated using an Optika B-290TB brightfield microscope, with analyses conducted via Fiji software (v1.54 f, NIH) across a minimum of five high-powered fields per animal.

### Masson’s trichrome staining and quantification of collagen content

Collagen deposition was assessed using Masson’s trichrome staining according to the manufacturer’s instructions (Trichrome Staining Kit, RWD, SCY-TRM1). Paraffin-embedded tissue sections were deparaffinized, rehydrated through graded alcohols, and stained to visualize collagen (blue), cytoplasm (red), and nuclei (dark). Brightfield images were acquired under identical magnification and exposure settings and analyzed using Fiji software version 1.54 f (National Institutes of Health, Bethesda, MD, USA) with a minimum of five high-powered fields per animal.

### TUNEL and immunofluorescence (IF) Staining

To determine the amount of cell death in lymphedema tissue, the TUNEL method was performed using 5 µm sections to label apoptotic DNA fragmentation in the tail sections using the Click-iT^TM^ Plus TUNEL Apoptosis Assay Kit (C10618, Thermo Fisher Scientific) combined with anti-LYVE-1 according to the manufacturer’s instructions. TUNEL-positive cells were imaged using a ZEISS Axio Imager 2 fluorescence microscope and quantified using Fiji software. For IF staining of tail tissues, sections were rehydrated, subjected to antigen retrieval, and then incubated overnight at 4 °C with anti-LYVE-1 (14-0443-80, Thermo Fisher Scientific) and anti-p16 (MA517142, Thermo Fisher Scientific).

For the VCX-treated cohort, additional staining for BCL-2 (ab194583, Abcam), F4/80 (MA191124, Thermo Fisher Scientific), CD4 (14-0041-82, Invitrogen), and LYVE-1 (PA1-16636, Thermo Fisher Scientific) was performed. Secondary antibodies were labeled with the fluorochromes Alexa Fluor 488 (A32723), Alexa Fluor 555 (A48263), Alexa Fluor 647 (A48289), and Alexa Fluor 647 (A32733) (Thermo Fisher Scientific). Nuclei were stained with 4′,6-diamidino-2-phenylindole (DAPI) by mounting the slides with ProLong™ Diamond Antifade Mountant with DAPI (P36962, Thermo Fisher Scientific). The slides were evaluated using a ZEISS Axio Imager 2 (ZEISS, Oberkochen, Germany) fluorescence microscope, respectively.

Fluorescence area and colocalization analyses were performed using Fiji software version 1.54 f (National Institutes of Health, Bethesda, MD, USA) with a minimum of ten high-powered fields per animal. For colocalization analysis, regions of interest (ROIs) corresponding to LYVE-1⁺ vessels were defined based on morphological features and fluorescence signal.

For in vitro experiments, HDLECs were exposed to ionizing radiation at doses of 5 or 10 Gy. Forty eight hours post-irradiation, cells were processed for IF staining using antibodies against p16 and BCL-2. MitoTracker™ Green (M7514, Thermo Fisher Scientific) was used to label mitochondria according to the manufacturer’s instructions. Colocalization analyses were performed to assess the spatial association between BCL-2 and mitochondrial signals. TUNEL staining and colocalization analyses were performed as described above. Co-expression analyses were conducted on a cell-by-cell basis to assess the expression patterns of p16, BCL-2, and TUNEL within individual cells. All fluorescence and colocalization analyses were quantified using Fiji software, as described above.

### Western blotting

To assess protein expression, protein lysates were prepared using NP40 lysis buffer (74385, Sigma) supplemented with protease and phosphatase inhibitors (78440, Thermo Fisher). Protein concentrations were determined using the Pierce BCA Protein Assay Kit (23225, Thermo Fisher Scientific), and measurements were taken with a FLUOstar Omega multi-plate reader (BMG Labtech, Ortenberg, Germany). Equal amounts of 20–30 µg of protein were loaded onto a Tris-glycine SDS-PAGE gel and separated by electrophoresis. Proteins were then transferred to nitrocellulose membranes using the Trans-Blot Turbo (1704150, Bio-Rad, Hercules, USA). Membranes were incubated overnight at 4 °C with primary antibodies diluted in 0.1% TBS-T, followed by a 1-h incubation with HRP-conjugated secondary antibodies. Protein bands were detected using Clarity Western ECL substrate (170-5060, Bio-Rad) and imaged with the UVP ChemStudio gel imaging system (76209-532, Analytikjena, Upland, CA, USA). Quantification of protein expression was performed using Vision Works software version 8.20 (Analytikjena). The following primary antibodies were used: anti- human/rat p53 (DO-1) (sc-126, Santa Cruz Biotechnology), anti- human CDKN2A/p16INK4a antibody (1D7D2A1) (ab201980, Abcam), anti- rat p16 INK4A (F-12) (sc-1661, Santa Cruz Biotechnology), recombinant anti- human/rat p21 antibody (EPR3993) (ab109199, Abcam), anti- human/rat BCL-2 antibody (ab194583, Abcam), anti-VEGF receptor 3 antibody (ab27278, Abcam), anti- human TNF-α (MA523720, Thermo Fisher Scientific), anti- human IFN-γ (3F1E3, Thermo Fisher Scientific), anti- GAPDH (6C5) (AM4300, Thermo Fisher Scientific), and anti- Tubulin (21255, Cell Signaling). Secondary antibodies of HRP goat anti-mouse IgG (926-80010) and HRP goat anti-rabbit IgG (926-80011) were purchased from LI-COR Biotechnology (Lincoln, NE, USA).

### Senescence associated secretory phenotype (SASP) measurement

To evaluate the changes in a variety of inflammatory and senescence-associated cytokines and chemokines called SASPs in humans, we quantified the levels of 39 different cytokines, chemokines, and growth factors in cancer-related lymphedema patient plasma samples, who underwent surgery, and/or RT with an MSD Cytokine Kit (K15209G-1, MesoScale Discovery Inc.). A Proteome Profiler Human Cytokine Array Kit (ARY005B, Cedarlane) was also used to evaluate the levels of 36 different cytokines and chemokines in HDLEC supernatants 48- and 72-h post-RT (5 Gy). A 4-Well multi-dish (#607544) with membranes (#898260) was used, and kit instructions were followed. The membranes were imaged using a Chemi Reagent Mix (#894287, 894288) in the UVP ChemStudio gel imaging system (Analytikjena, Upland, CA, USA) and quantified using Vision Works software version 8.20 (Analytikjena). The ultimate levels of cytokines in both media supernatants and patient samples were normalized to normal controls and used for making heat maps and a volcano plot using GraphPad Prism version 10.2.3.

### Statistical analysis

All graphs and statistical analyses were generated using GraphPad Prism version 10.2.3 by a blinded investigator. When necessary, the Shapiro–Wilk test was used for normalization, and appropriate statistical tests were applied accordingly. Pearson correlation analysis was used to assess correlations among human plasma analytes, and differences between groups were evaluated using one-way ANOVA followed by appropriate post hoc tests. For the cell death assay and mouse data, differences between groups were identified using two-way ANOVA followed by Šídák’s post hoc test. For other in vitro data, including clonogenicity assay and western blotting data, statistical significance was determined using one-way ANOVA followed by Tukey’s post hoc test or an unpaired t-test. Data are presented as mean ± standard error of the mean (SEM). Each test was performed as three replicates (*n* = 3) or more, and p-values lower than 0.05 were considered statistically significant. Homogeneity of variance between groups was assessed and found to be comparable.

## Supplementary information

Supplementary Figures 1-20

Uncropped western blots

## Data Availability

The data generated during this study were included in the article and its supplementary files. They are also available from the corresponding author upon reasonable request.
