# Supplementary material for: Radiation induces senescence in lymphatic endothelial cells (LECs) and murine tail lymphedema tissue, contributing to lymphedema progression
Source: Cell Death Dis. 2026 Jun 10;17(1):733. doi: 10.1038/s41419-026-08955-z (PMC13478601; doi:10.1038/s41419-026-08955-z)
Supplement: Supplementary file 1 [file 41419_2026_8955_MOESM1_ESM.pdf]

Supplementary Information

Radiation induces senescence in lymphatic endothelial cells (LECs) and murine tail lymphedema tissue, contributing to lymphedema progression

Samaneh Safarpour et al.
Corresponding author: Spencer B. Gibson, sgibson2@ualberta.ca

This PDF file includes:

Supplementary Figures and Table

Supplemental Figure 1

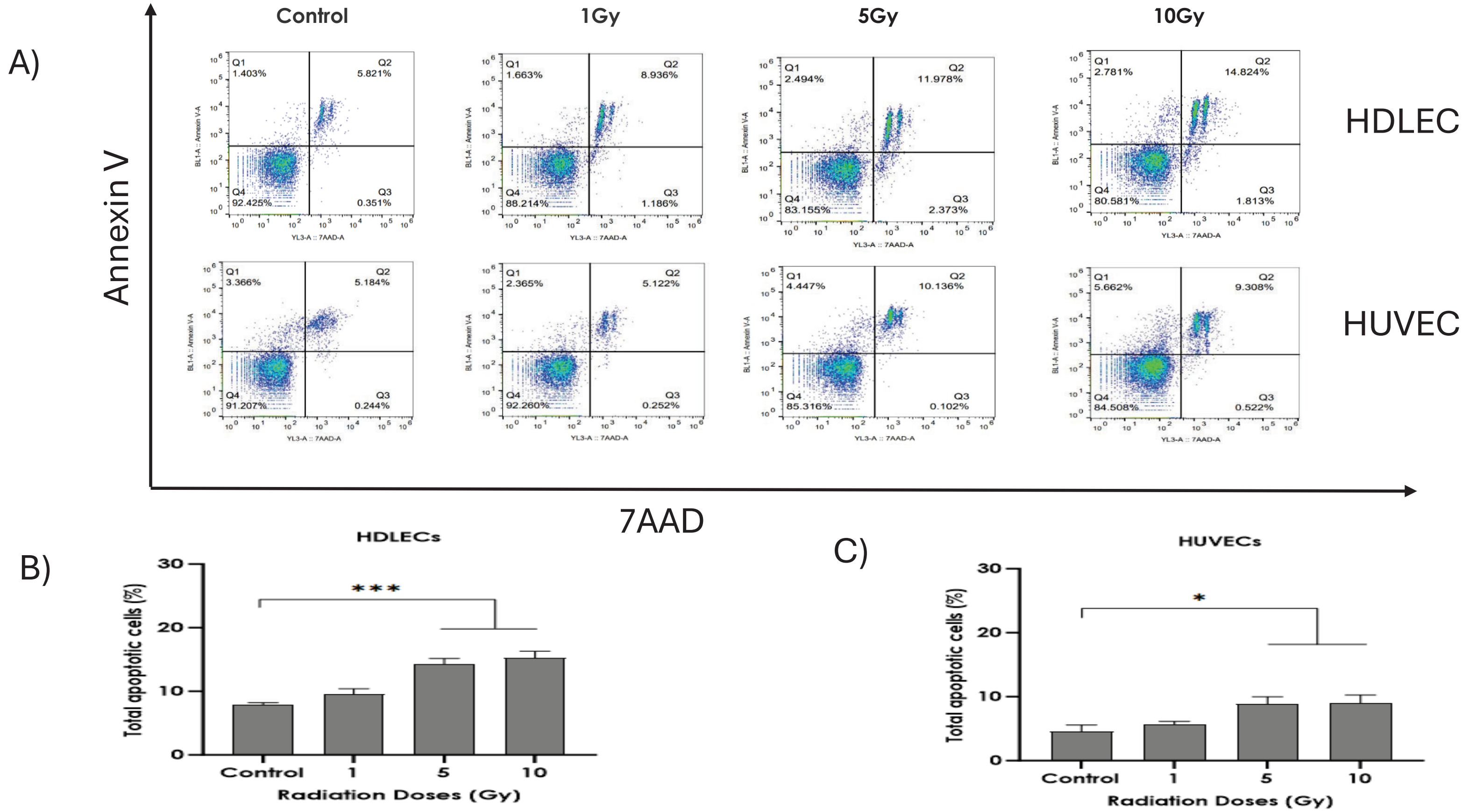

Supplemental Figure 1. Radiation induces apoptosis in endothelial cells in a dose-independent manner. Annexin-V/7AAD assay was performed on HDLECs and HUVECs after radiation treatment (1-10Gy). A) A schematic view of the graphs from the Attune NxT flow cytometer and FlowJo related to radiation treatment shows percentages of total apoptotic cells (sum of annexin-V+/7-AAD− and annexin-V+/7-AAD+) after treatment. The total apoptotic cell death was determined for B) HDLECs and C) HUVECs with different doses of radiation (1, 5, and 10Gy) in three independent experiments. Data are presented as mean ± SEM, *P < 0.05 and ***P < 0.001.

Supplemental Figure 2

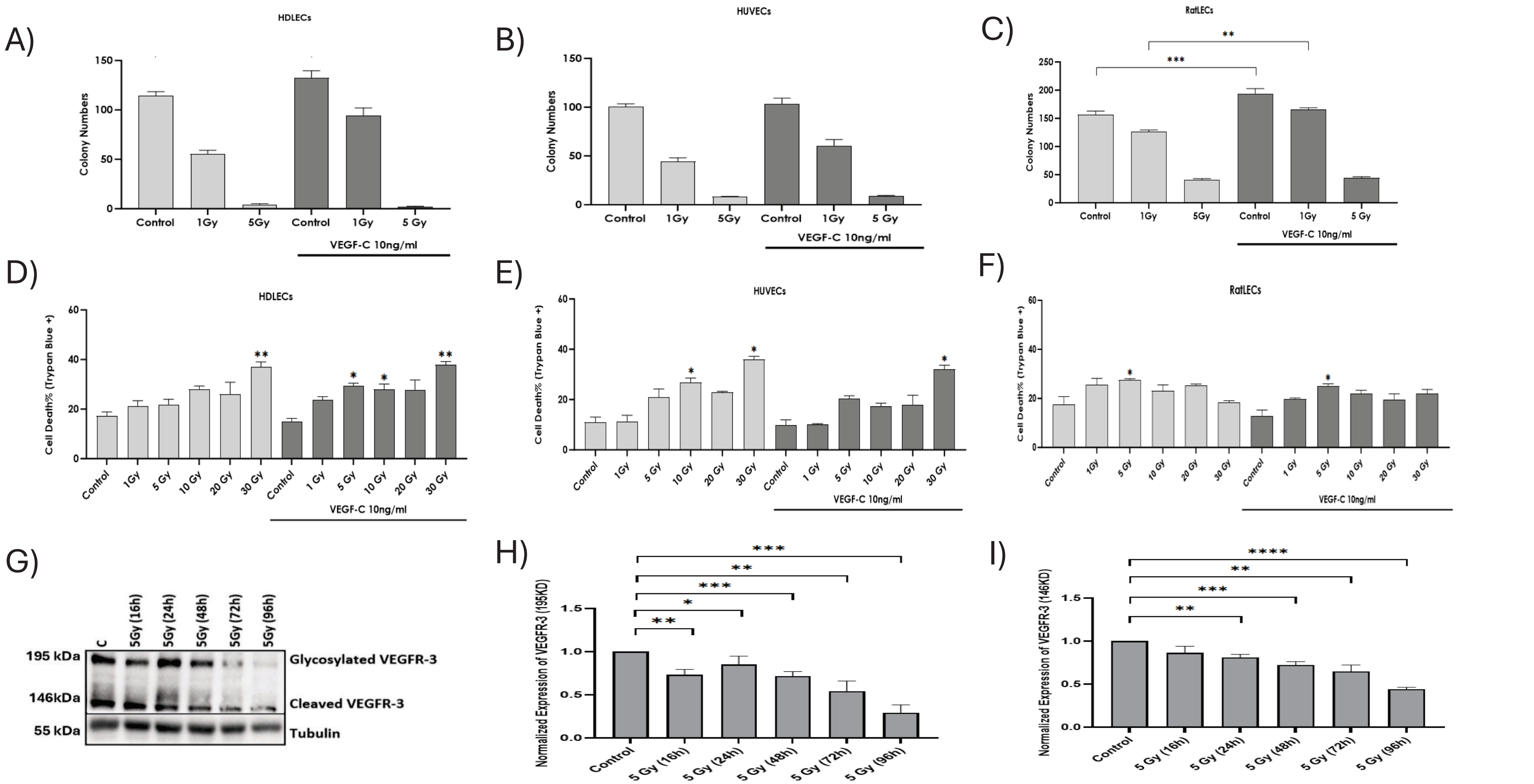

Supplemental Figure 2. VEGF-C increases lymphatic endothelial cell growth but fails to rescue cells from growth inhibition and death under RT stress. A) HDLECs, B) HUVECs, and C) ratLECs were treated with 10ng/ml of VEGF-C for 1hour in combination with 1 and 5Gy of radiation. The cells were then incubated in an incubator for 7-14 days, and a colony-forming assay was conducted. D-F) A trypan blue exclusion assay was performed on the same cells following 48h of radiation treatment in the presence or absence of VEGF-C. G) Representative western blot images for two subunits of VEGFR-3 in HDLECs were shown after being treated with 5Gy of radiation doses over a 96-hour time course. Tubulin was a loading control. H-I) Densitometry of the VEGFR-3 subunits was performed on three independent experiments. Data was presented as mean ± SEM, $*P < 0.05$, $**P < 0.01$, $***P < 0.001$, and $****P<0.0001$.

Supplemental Figure 3

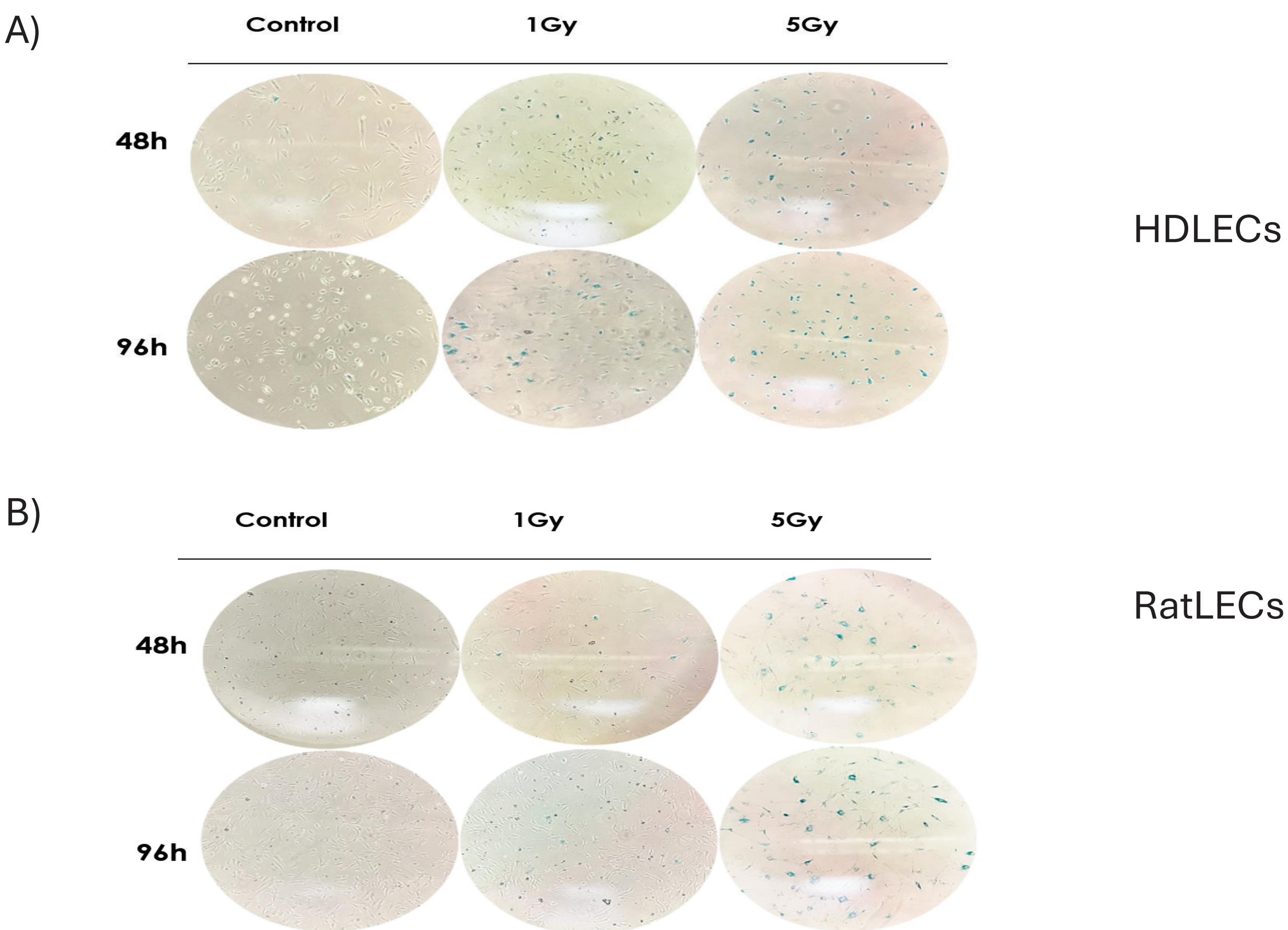

Supplemental Figure 3. Images of SA-beta-galactosidase positive lymphatic cells after radiation treatment. A) HDLECs and B) RatLECs were treated with 1 and 5Gy radiation and incubated for 48 and 96 hours. Senescent cells were detected by a SA-βgal assay. Blue-stained cells were examined under a light microscope, and representative images are presented above.

Supplemental Figure 4

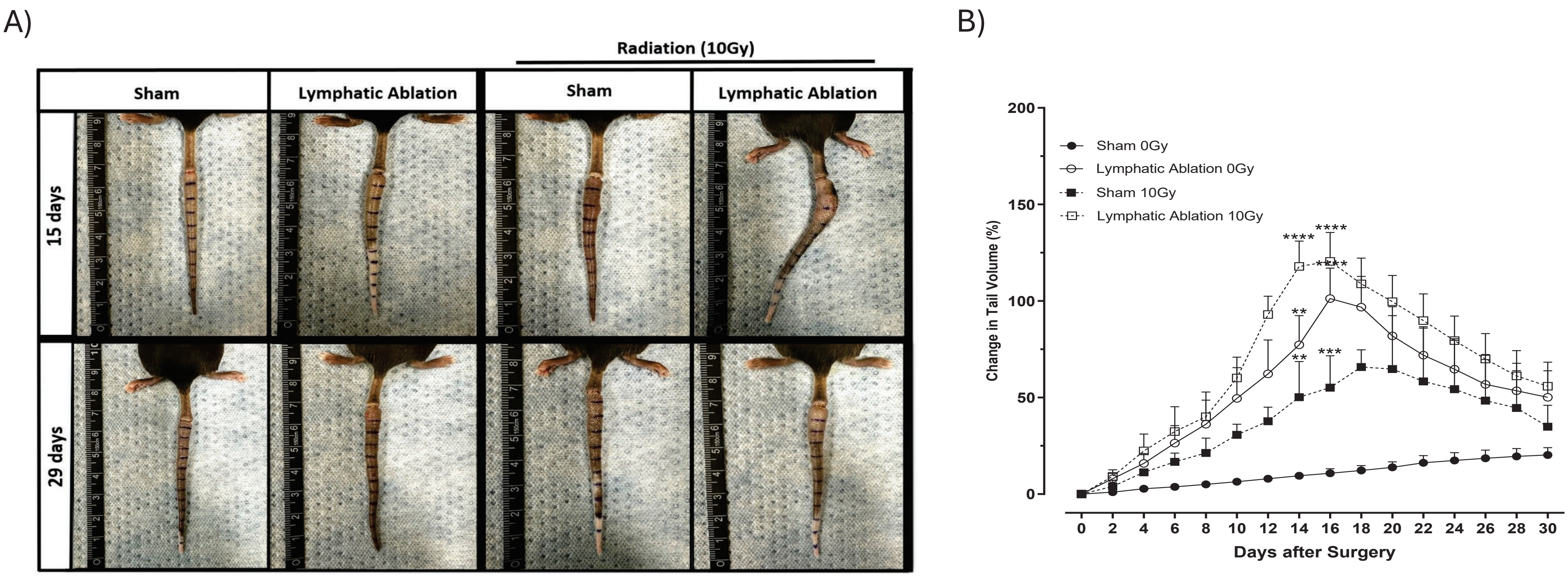

Supplemental Figure 4. Radiation induced tail lymphedema. A) Representative image of tails in the sham, lymphatic ablation, with or without radiation (10Gy). B) Graph indicates the changes in tail volume over 30 days post-surgery. Data are presented as mean ± SEM of five independent experiments. **$P < 0.01$, ***$P < 0.001$, and ****$P<0.0001$.

Supplemental Figure 5

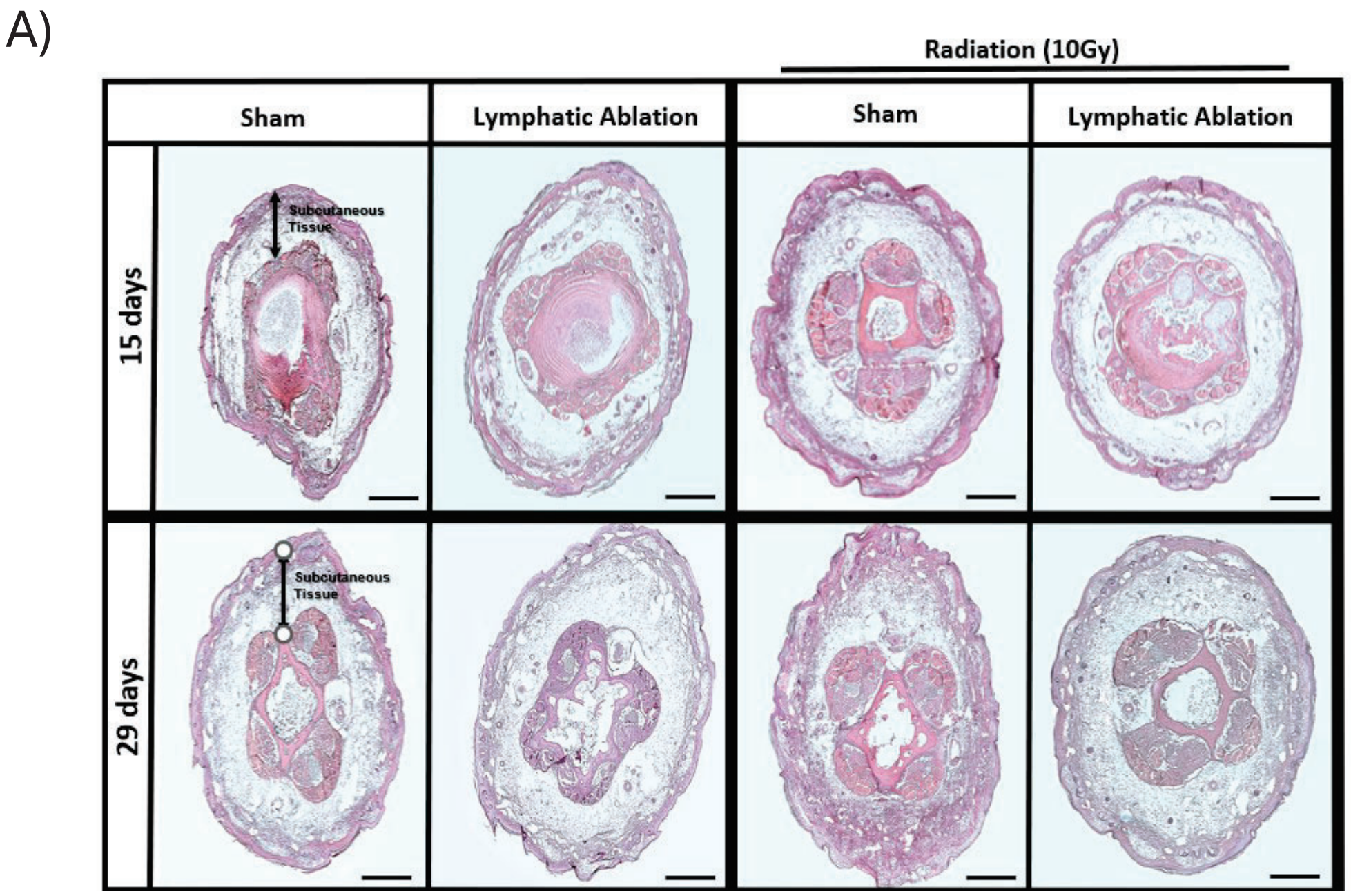

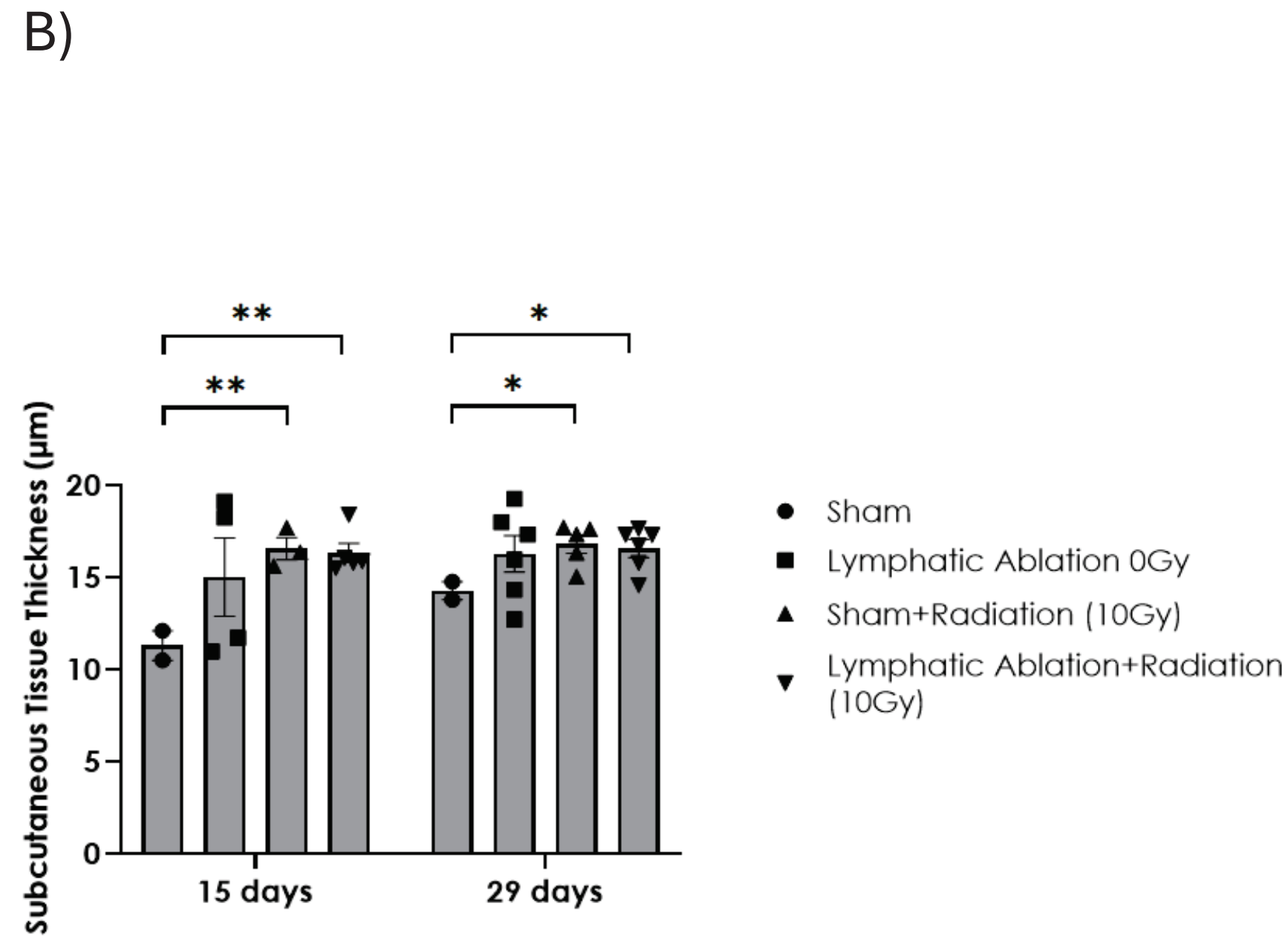

Supplemental Figure 5. Radiation increases subcutaneous tissue thickness in the tail lymphedema model. A) Representative Hematoxylin & Eosin staining from cross-sections of tail tissues indicates lymphatic ablation or sham surgery in the presence or absence of radiation. Subcutaneous tissue thickness 15- and 29-day post RT in sham or lymphedema ablation was calculated. The double-sided black arrow shows subcutaneous tissues. B) Quantification of subcutaneous tissue thickness after 15 and 29 days of RT was calculated from five independent experiments. Data represented as mean ± SEM. *P<0.05, and **P < 0.01. Scale bar: 10 µm.

Supplemental Figure 6

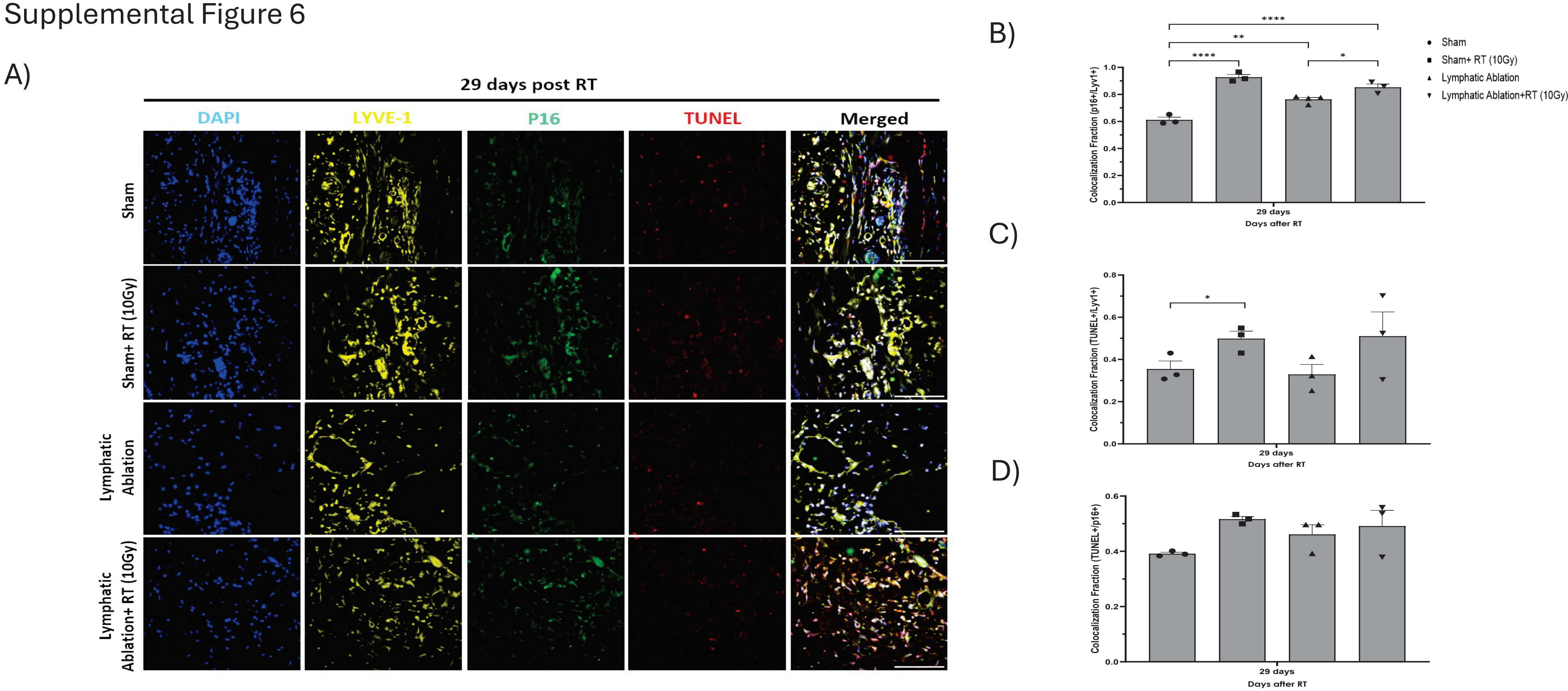

Supplemental Figure 6. Senescence marker p16 and cell death levels were increased 29 days post RT in the tail lymphedema model. A) Representative immunofluorescence staining of LYVE-1-positive lymphatic endothelial cells (yellow) and senescence marker p16 (green), apoptosis (TUNEL) (red) in tail tissues from lymphedema mice, 29 days post-RT, shows higher colocalization of p16+ and LYVE-1+ in sham+10Gy and lymphatic ablation+10Gy, and TUNEL+ and LYVE-1+ in sham+10Gy. DAPI stains the nucleus (blue). Scatter plots are showing B) p16+ LYVE-1+ colocalization fraction, C) TUNEL+ LYVE-1+ colocalization fraction, and D) p16+ TUNEL+ colocalization fraction and comparing the groups. n=3, data are presented as mean ± SEM, *P < 0.05, **P < 0.01, and ****P<0.0001. Scale bars: 100 µm. LYVE-1: lymphatic vessel endothelial hyaluronan receptor-1; TUNEL: terminal deoxynucleotidyl transferase dUTP nick end labeling; DAPI: 4′,6-diamidino-2-phenylindole.

Supplemental Figure 7

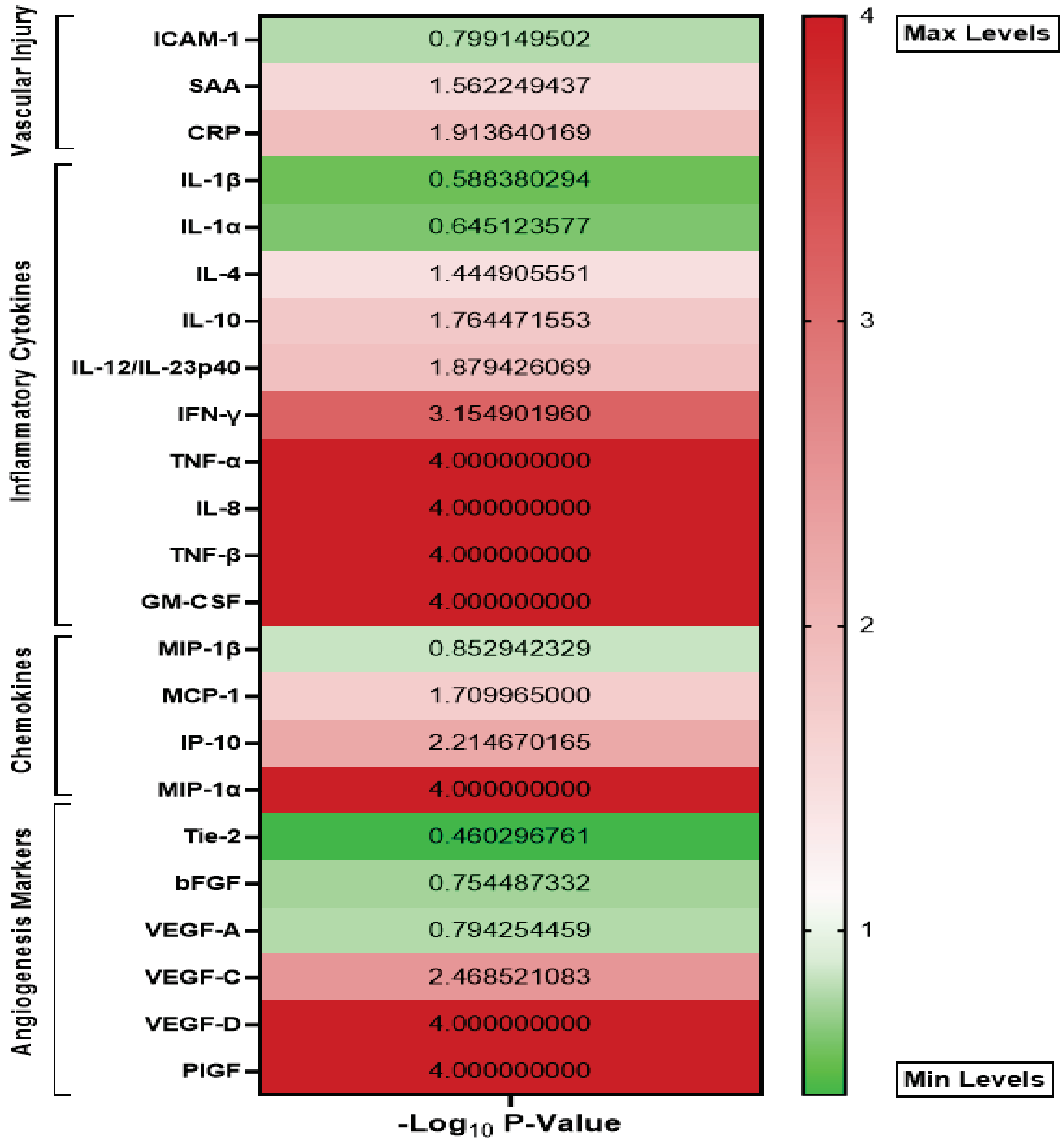

Supplemental Figure 7. Heat map of SASP signature in lymphedema patients. Heat map represents differential levels of senescence-associated secretory phenotypes (SASPs), including vascular injury factors, inflammatory cytokines, chemokines, and angiogenic and lymphangiogenic markers in plasma samples of lymphedema patients who underwent surgery and/or RT. These inflammatory factors were determined by a MesoScale assay. The data is normalized to normal controls. The red colors represent high values while green colors show lower values of markers relative to the control.

Supplemental Figure 8

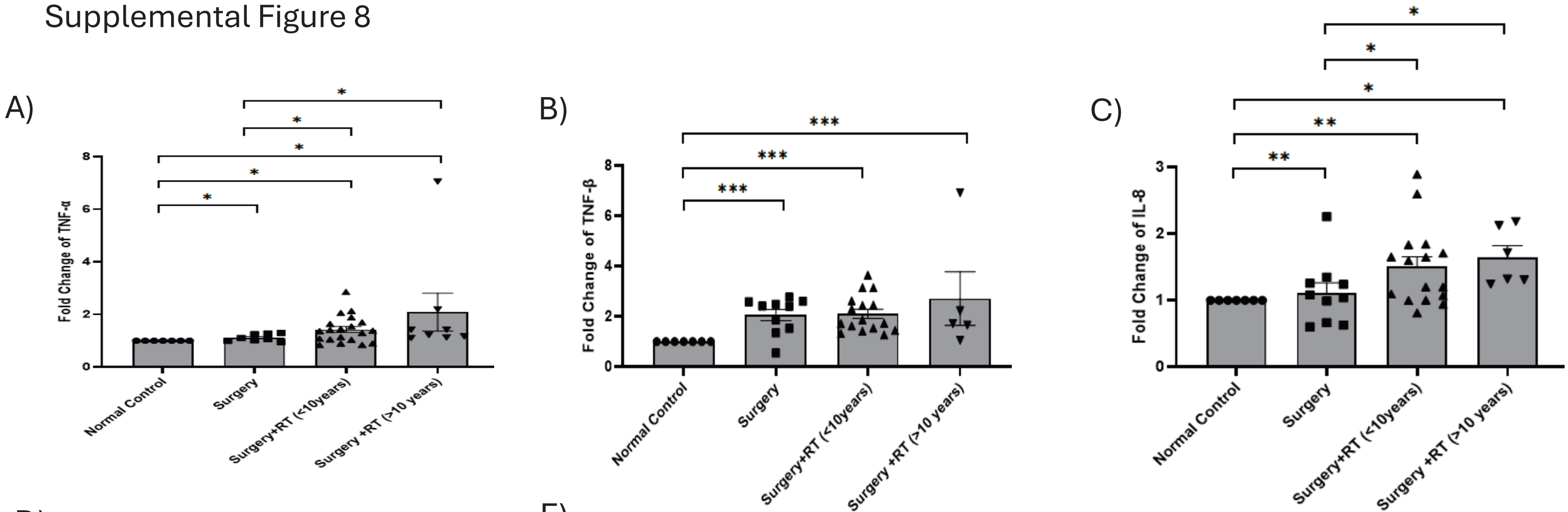

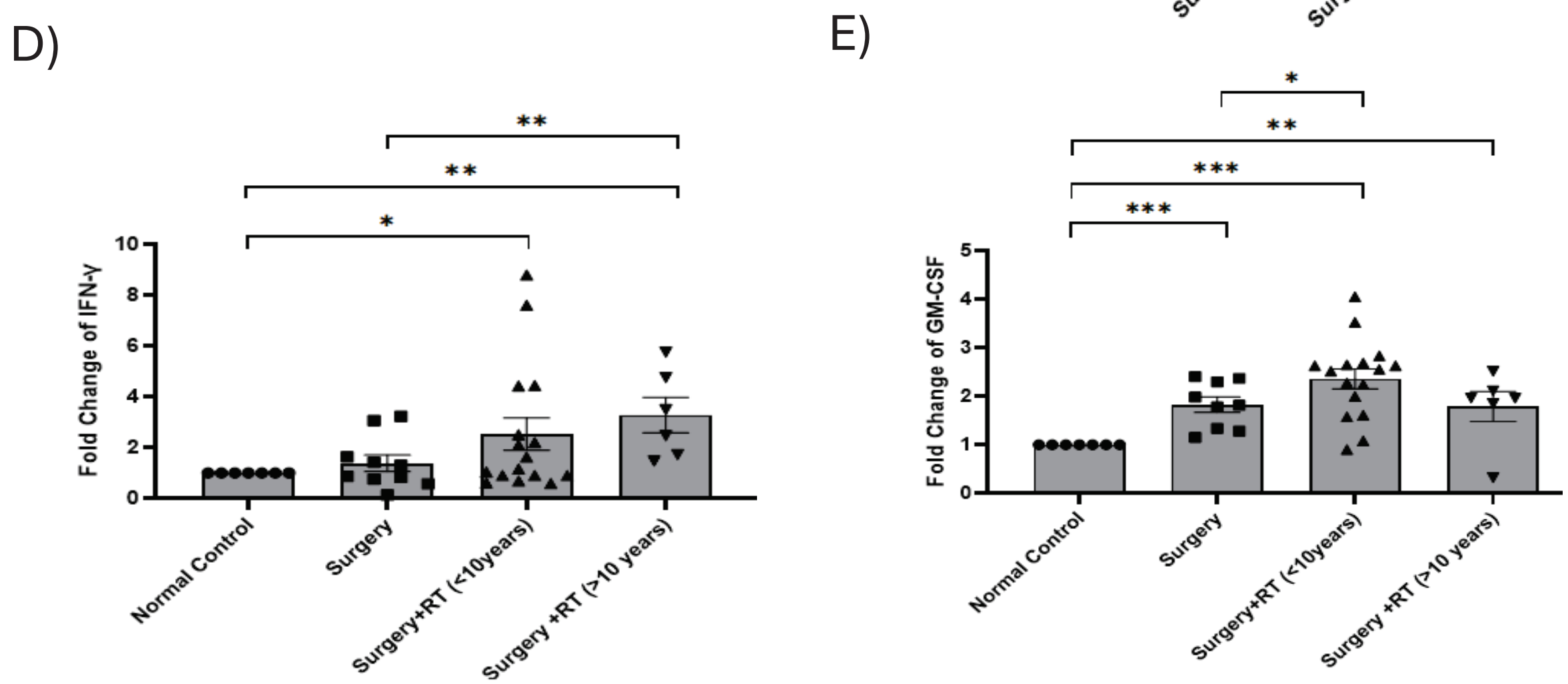

Supplemental Figure 8. Expression levels of inflammatory factors in lymphedema patients are not affected by the amount of time elapsed from receiving RT. Inflammatory cytokines were determined by a MesoScale assay. The fold change was compared to non-lymphedema controls. Bar graphs represent fold increase in inflammatory cytokines; A) TNF-α, B) TNF-β, C) IL-8, D) IFN-γ, and E) GM-CSF in plasma samples of lymphedema patients who underwent surgery, or surgery+RT (<10 years or >10 years), compared to normal controls. The dots indicate the number of individuals in each group. Data are presented as mean ± SEM, $^{*}P < 0.05$, $^{**}P < 0.01$, and $^{***}P < 0.001$.

Supplemental Figure 9

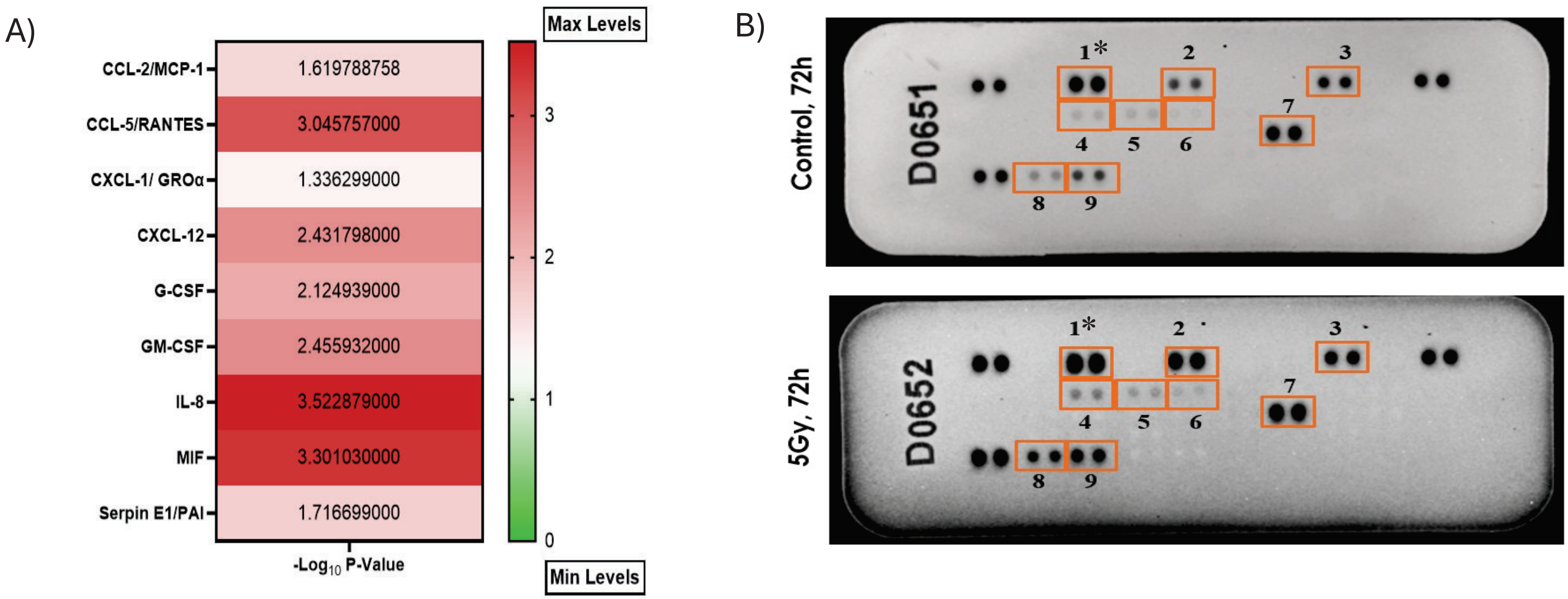

Supplemental Figure 9. Levels of inflammatory cytokines increased in radiation-treated HDLECs' supernatants. The fold changes in 36 different cytokines and chemokines were determined in HDLECs' supernatants post treatment compared to non-treated cells using a Proteome Profiler Human Cytokine Array Kit. A) The fold changes were depicted by heat maps representing differential levels of inflammatory cytokines and chemokines 72-hours post radiation treatment compared to control. The red colors represent high values, while the green colors show lower values of markers relative to the control. B) Representative membranes from HDLECs' supernatant incubated with 5Gy of RT compared to the control from two independent experiments. *1: CCL-2/MCP-1, 2: CCL-5/RANTES, 3: CXCL-1/ GROα, 4: CXCL-12, 5: G-CSF, 6: GM-CSF, 7: IL-8, 8: MIF, and 9: Serpin E1/PAI.

Supplemental Figure 10

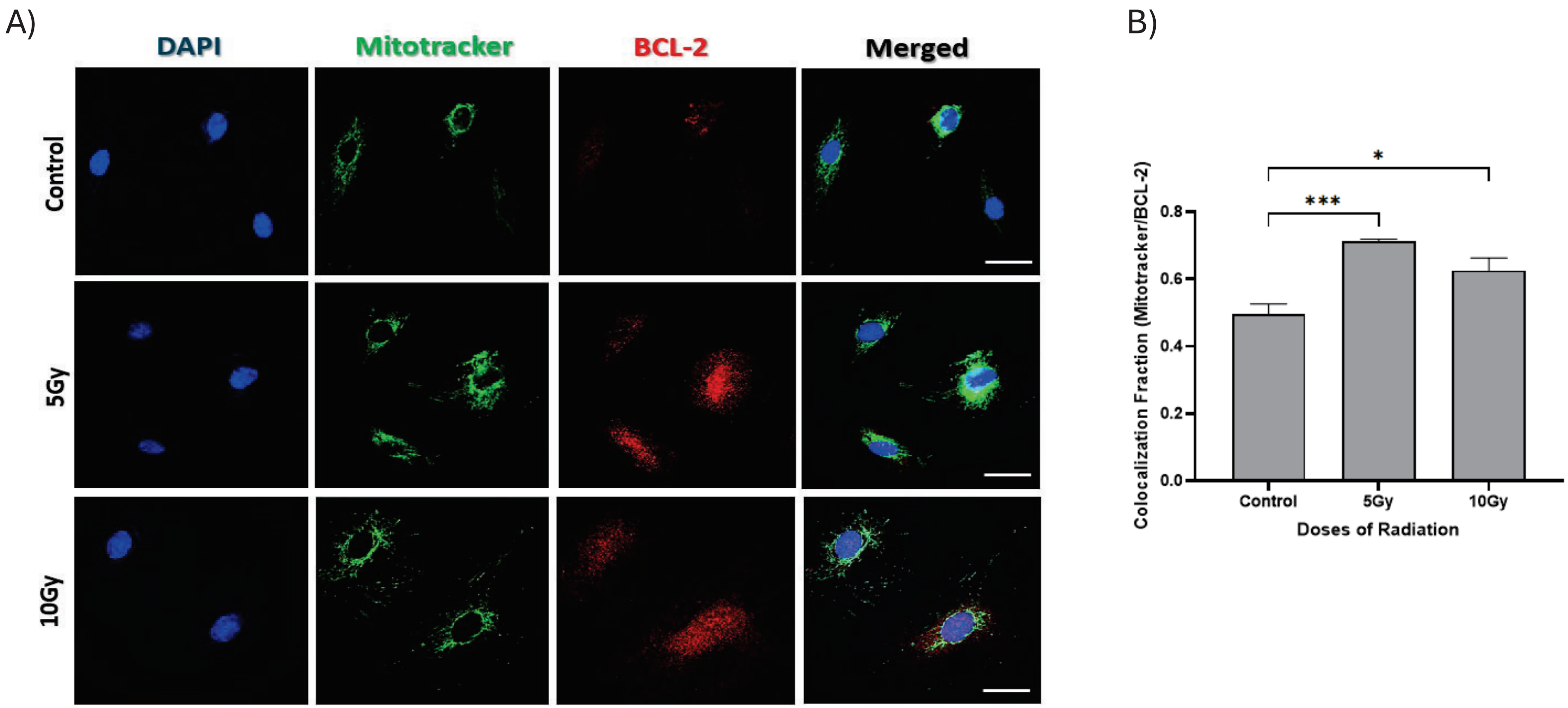

Supplemental Figure 10. Radiation enhances BCL-2 localization to mitochondria in HDLECs. A) Representative immunofluorescence images of HDLECs stained with DAPI (blue), MitoTracker (green; mitochondria), and BCL-2 (red). Merged images demonstrate the colocalization of BCL-2 with mitochondrial structures. Cells were analyzed under control conditions and following exposure to 5Gy and 10Gy radiation. B) Quantification of BCL-2 colocalization with mitochondria, expressed as colocalization fraction. Experiments represent 6-8 independent repeats, and error bars represent mean ± SEM. *P < 0.05 and ***P < 0.001. Scale bar: 50μm.

Supplemental Figure 11

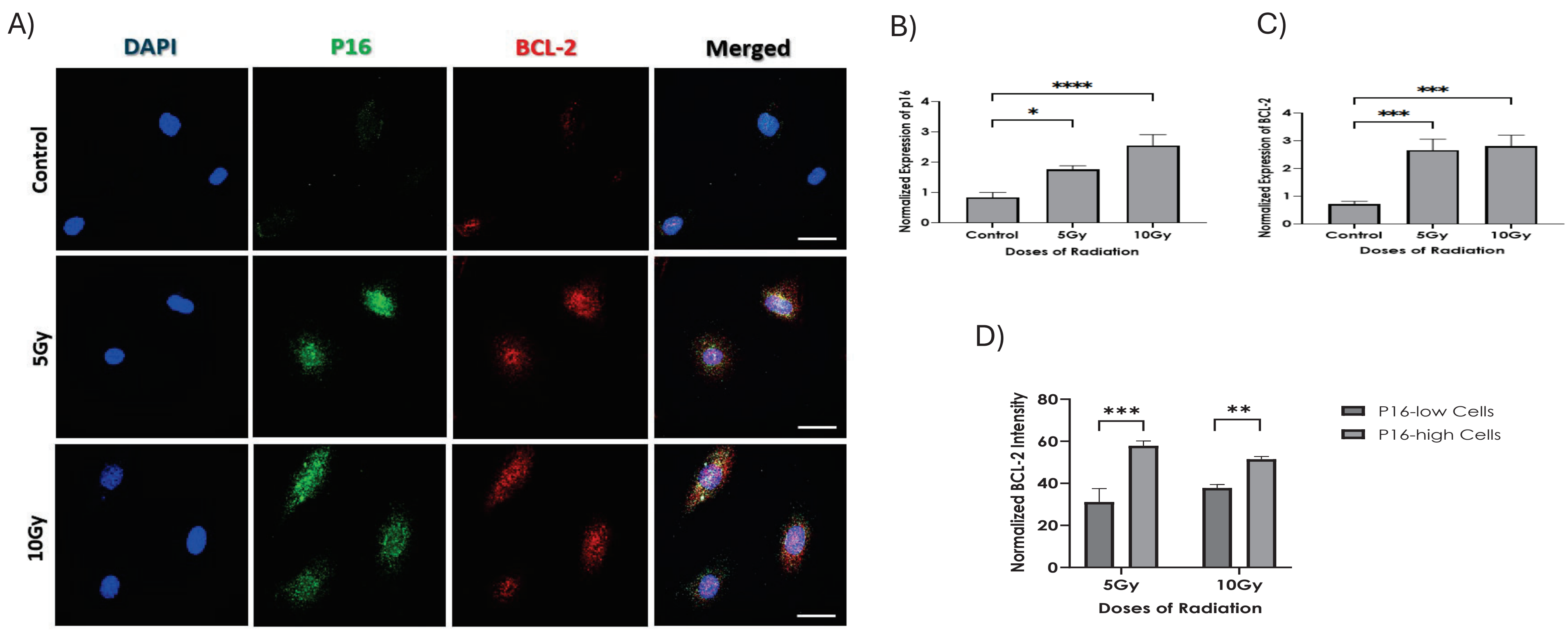

Supplemental Figure 11. Radiation induces upregulation of senescence-associated p16 and BCL-2 in HDLECs. A) Representative immunofluorescence images of HDLECs stained for DAPI (blue), p16 (green; senescence marker), and BCL-2 (red). Merged images illustrate co-expression patterns under control conditions and following exposure to 5Gy and 10Gy radiation. Quantification of B) p16 expression and C) BCL-2 expression in irradiated cells compared to control. D) Comparison of BCL-2 expression between p16-low and p16-high cell populations. Experiments represent 6-8 independent repeats, and error bars represent mean ± SEM. *P < 0.05, ***P < 0.001, and ****P < 0.0001. Scale bar: 50μm.

Supplemental Figure 12

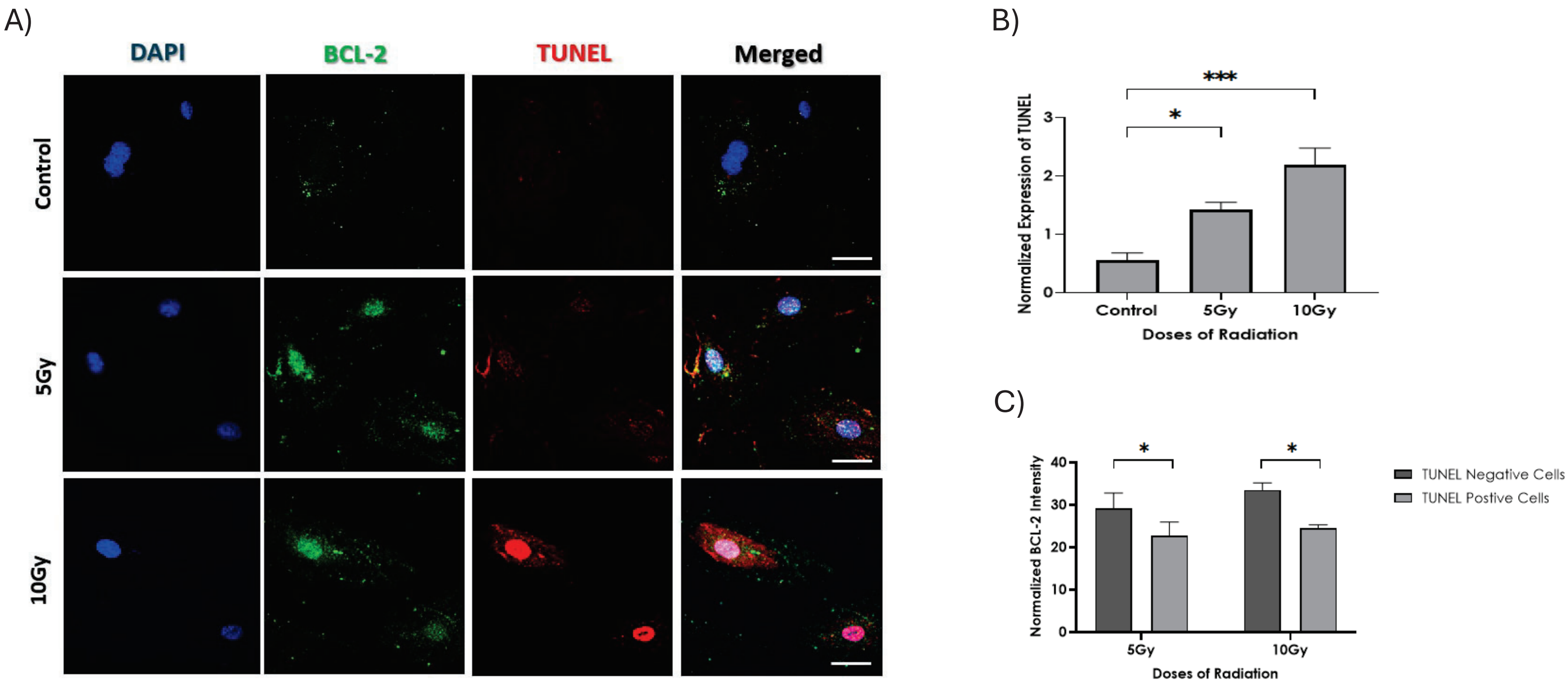

Supplemental Figure 12. Radiation-induced apoptosis in HDLECs is associated with differential BCL-2 expression. A) Representative immunofluorescence images of HDLECs stained with DAPI (blue), BCL-2 (green), and TUNEL (red; apoptotic cells). Merged images illustrate the relationship between BCL-2 expression and apoptosis under control conditions and following exposure to 5Gy and 10Gy radiation. B) Quantification of TUNEL-positive cells following radiation and C) BCL-2 intensity in TUNEL-negative and TUNEL-positive cells. Experiments represent 6-8 independent repeats, and error bars represent mean ± SEM. $^*P < 0.05$ and $^{***}P < 0.001$. Scale bar: 50μm.

Supplemental Figure 13

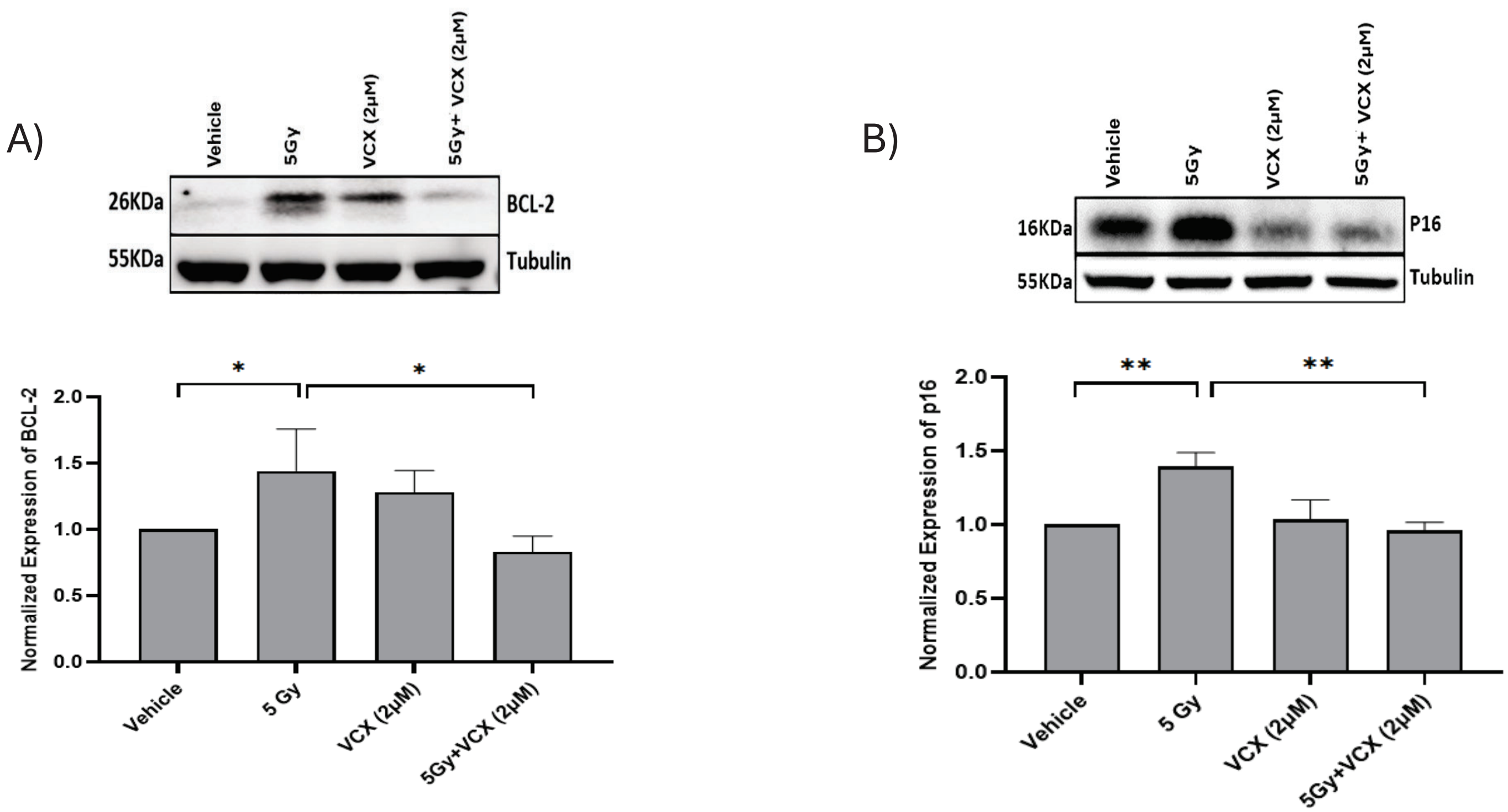

Supplemental Figure 13. Expression levels of BCL-2 and p16 were reduced after VCX treatment in RT-treated HDLECs. BCL-2 and p16 protein expression levels were determined by western blotting. Tubulin was used as a loading control. Representative western blot images for A) BCL-2 and B) p16 after treating HDLECs with 5Gy of radiation dose for 48h and then VCX for 24h. All experiments were repeated three times independently. Error bars represent mean ± SEM, $^{*}P < 0.05$ and $^{**}P < 0.01$.

Supplemental Figure 14

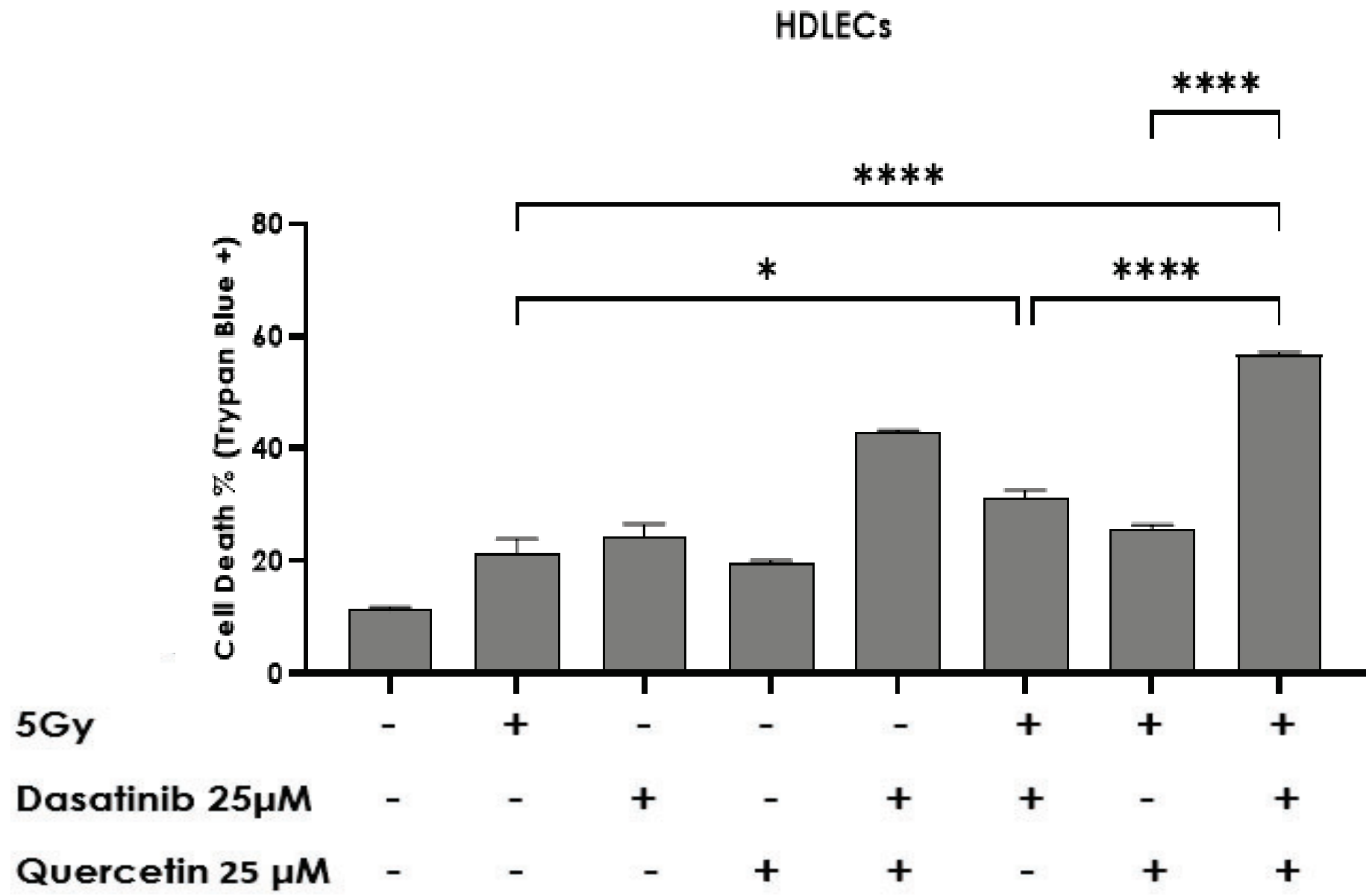

Supplemental Figure 14. Senolytic agents, dasatinib (D) and quercetin (Q), induced cell death in RT- treated HDLECs. Bar graph represents the percentage of cell death by trypan blue exclusion assay after treatment of HDLECs with 5Gy of radiation dose for 48h, and then 25µM of D and 25µM of Q for 24h. All experiments were repeated three times independently. Data are presented as mean ± SEM, *P<0.05 and ****P<0.0001.

Supplemental Figure 15

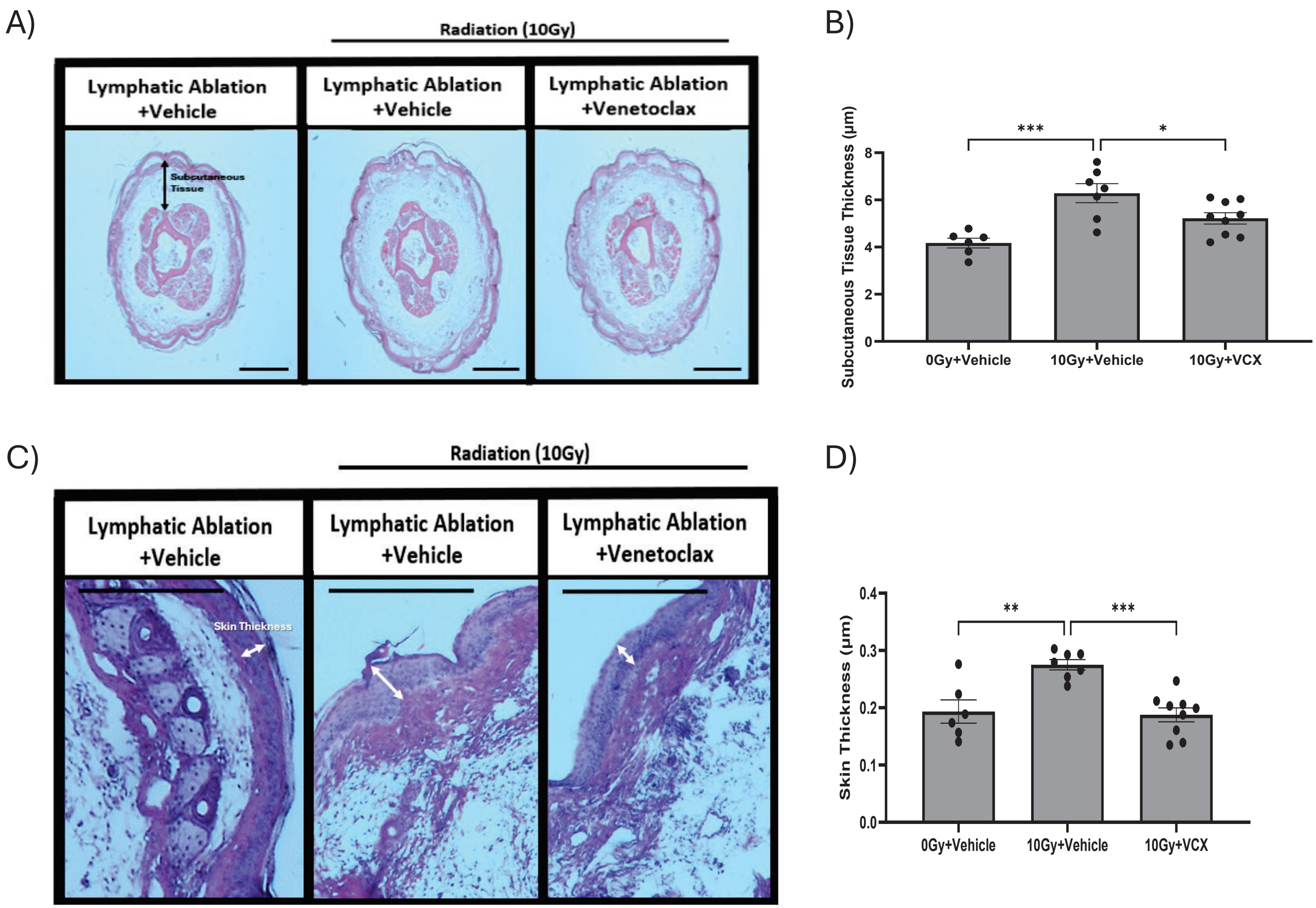

Supplemental Figure 15. VCX attenuates radiation-induced tissue thickening in a murine tail lymphedema model. A) Representative histological H&E-stained cross-sections of mouse tail tissues showing subcutaneous tissue thickness following lymphatic ablation under control conditions (0Gy+vehicle), radiation (10Gy+vehicle), and radiation combined with VCX (10Gy+VCX). Scale bar: 10μm, B) Quantification of subcutaneous tissue thickness (μm), following radiation and VCX treatment. C) Representative histological images of skin layers showing skin thickness after radiation and VCX treatments. D) Quantification of skin thickness (μm) across experimental groups. Experiments represent 6-8 independent repeats, and error bars represent mean ± SEM. *$P < 0.05$, **$P < 0.01$, and ***$P < 0.001$. Scale bar: 20 μm.

Supplemental Figure 16

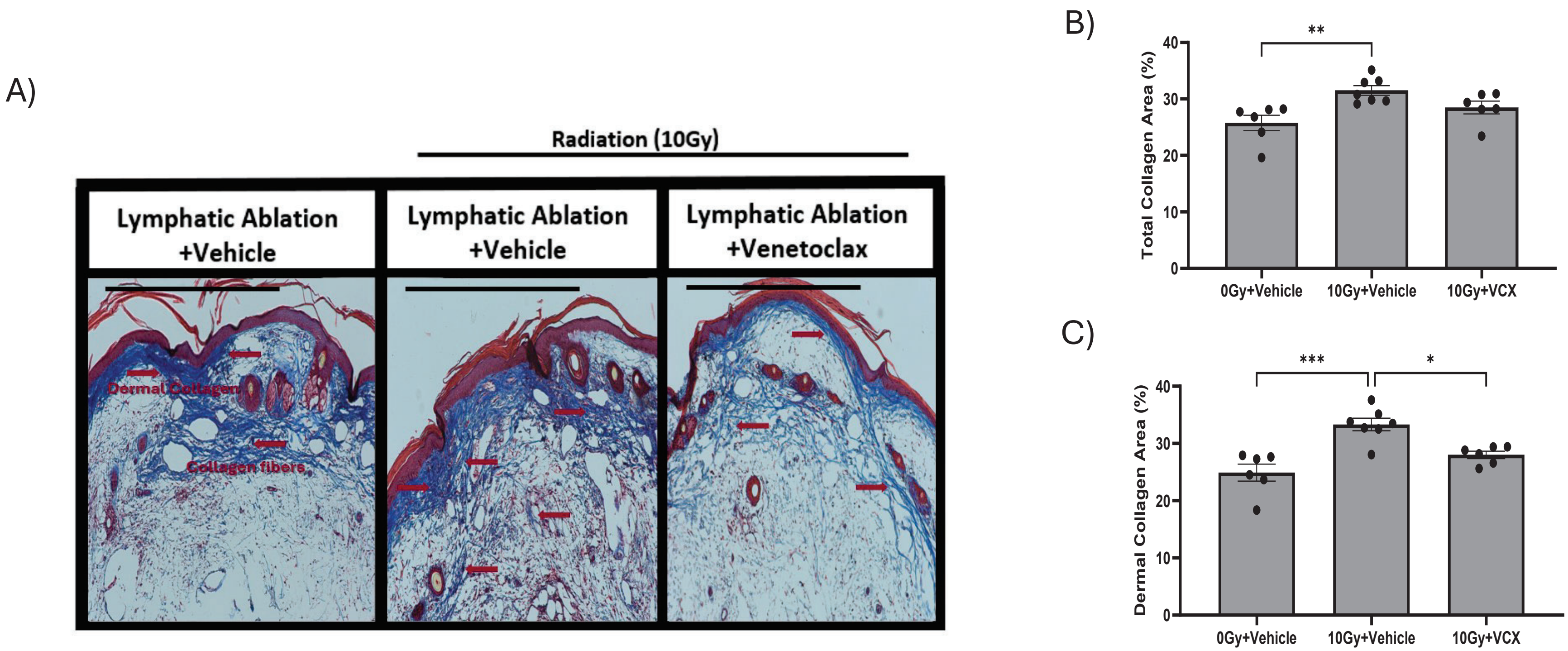

Supplemental Figure 16. VCX reduces radiation-induced collagen deposition in a murine tail lymphedema model. A) Representative Masson's trichrome-stained sections of mouse tail tissues following lymphatic ablation under control conditions (0Gy+vehicle), radiation (10Gy+vehicle), and radiation combined with VCX (10Gy+VCX). Collagen fibers are stained blue. B) Quantification of total collagen area (%) across experimental groups. C) Quantification of dermal collagen area (%) following radiation and VCX treatment. Experiments represent 6-8 independent repeats, and error bars represent mean ± SEM. *P < 0.05, **P < 0.01, and ***P < 0.001. Scale bar: 20μm.

Supplemental Figure 17

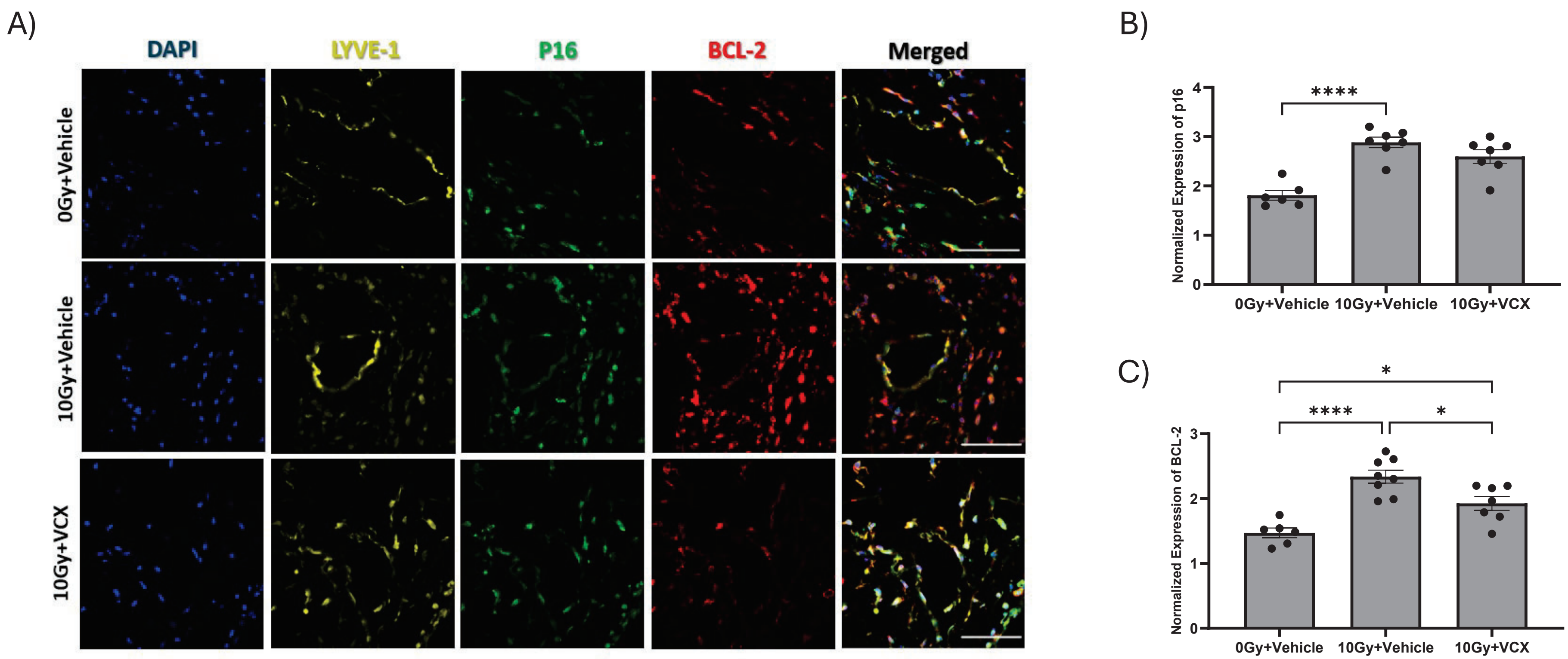

Supplemental Figure 17. Radiation-induced BCL-2 overexpression is attenuated in lymphatic endothelial cells by VCX treatment in a murine tail lymphedema model. A) Representative immunofluorescence images of tail tissue sections stained for DAPI (blue), LYVE-1 (yellow), p16 (green), and BCL-2 (red). Groups include control (0Gy+vehicle), radiation (10Gy+vehicle), and radiation (10Gy) combined with VCX (10Gy+VCX). B) Quantification of p16 expression in LYVE-1-positive cells. C) Quantification of BCL-2 expression in LYVE-1-positive cells. Experiments represent 6-8 independent repeats, and error bars represent mean ± SEM. *P < 0.05 and ****P < 0.0001. Scale bar: 50μm. LYVE-1: lymphatic vessel endothelial hyaluronan receptor-1; p16: senescence marker; BCL-2: anti-apoptotic protein; and DAPI: 4′, 6-diamidino-2-phenylindole.

Supplemental Figure 18

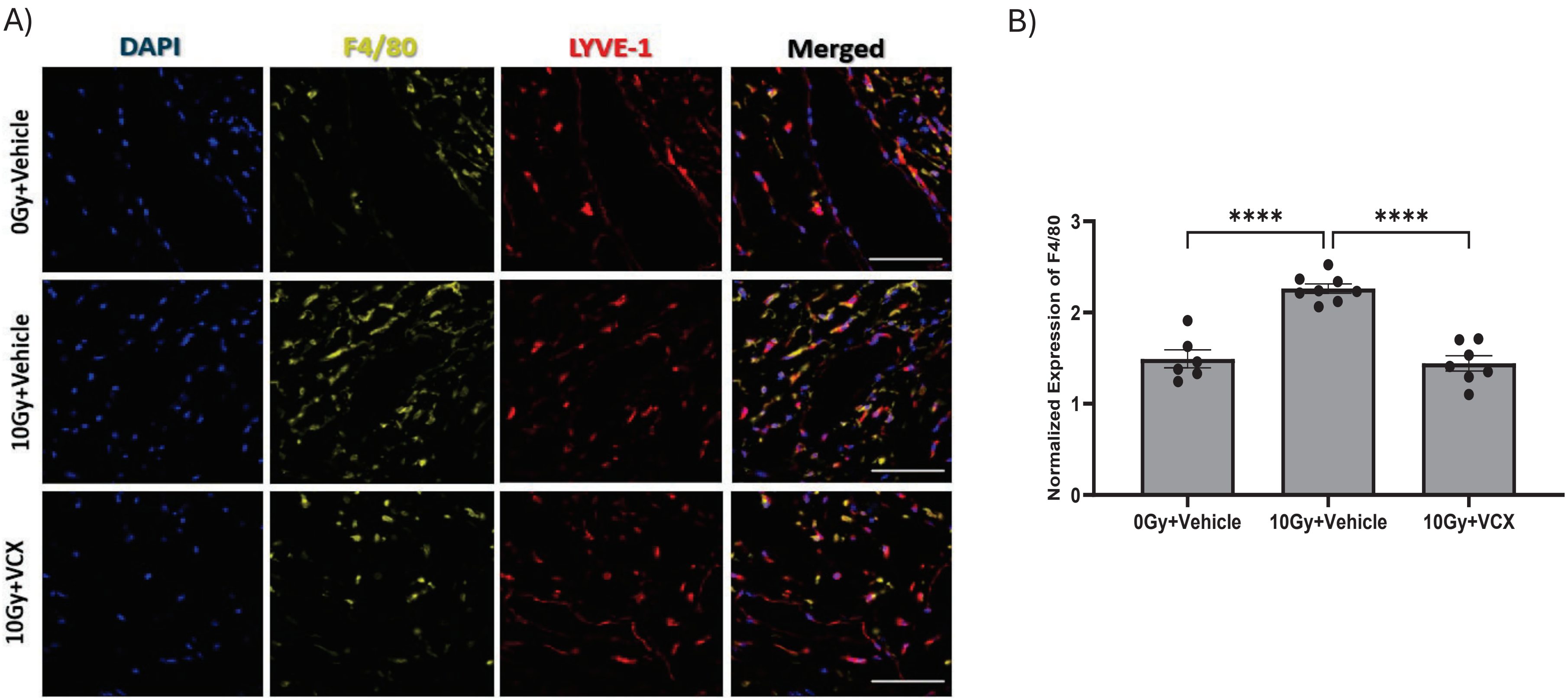

Supplemental Figure 18. Radiation-induced macrophage infiltration is attenuated by VCX in a murine tail lymphedema model. A) Representative immunofluorescence images of tail tissue sections stained for DAPI (blue), F4/80 (yellow), and LYVE-1 (red). Merged images show the spatial distribution of macrophages relative to lymphatic vessels. Experimental groups include control (0Gy+ vehicle), radiation (10Gy+vehicle), and radiation (10Gy) combined with VCX (10Gy+VCX). B) Quantification of F4/80 expression in tail tissues. Experiments represent 6-8 independent repeats, and error bars represent mean ± SEM, ****P < 0.0001. Scale bar: 50μm. LYVE-1: lymphatic vessel endothelial hyaluronan receptor-1; F4/80: macrophage marker; and DAPI: 4′, 6-diamidino-2-phenylindole.

Supplemental Figure 19

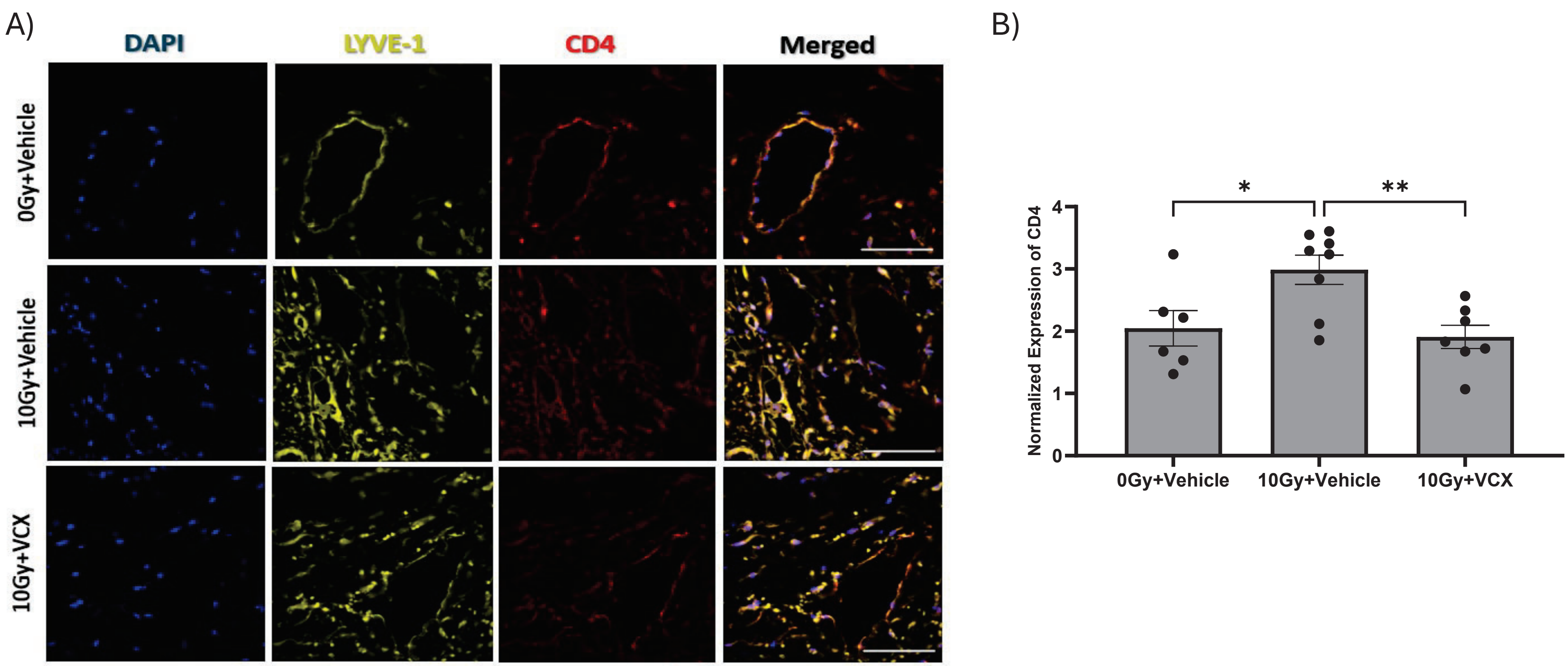

Supplemental Figure 19. Radiation-induced CD4⁺ T cell infiltration is reduced by VCX in a murine tail lymphedema model. A) Representative immunofluorescence images of tail tissue sections stained for DAPI (blue), LYVE-1 (yellow), and CD4 (red). Merged images demonstrate the localization of $CD4^+$ cells in proximity to lymphatic vessels. Groups include control (0Gy+vehicle), radiation (10Gy+vehicle), and radiation (10Gy) combined with VCX (10Gy+VCX). B) Quantification of CD4 expression in tail tissues. Experiments represent 6-8 independent repeats, and error bars represent mean ± SEM, $^*P < 0.05$ and $^{**}P < 0.01$. Scale bar: 50μm. LYVE-1: lymphatic vessel endothelial hyaluronan receptor-1; CD4: T helper cell marker; and DAPI: 4′, 6-diamidino-2-phenylindole.

Supplemental Figure 20

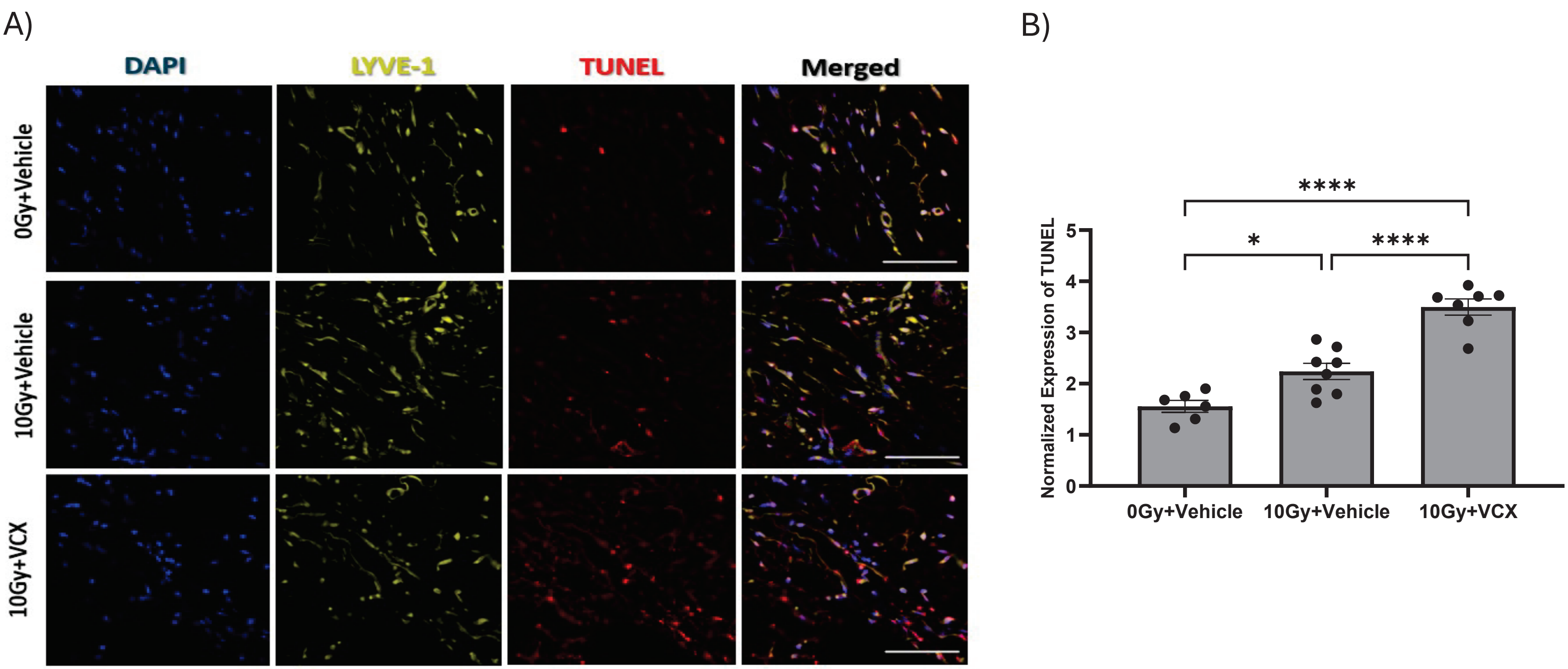

Supplemental Figure 20. VCX enhances apoptosis in lymphatic endothelial cells following radiation in a murine tail lymphedema model. A) Representative immunofluorescence images of tail tissue sections stained for DAPI (blue), LYVE-1 (yellow), and TUNEL (red). Merged images show apoptotic cells in relation to lymphatic vessels. Experimental groups include control (0Gy+vehicle), radiation (10Gy+vehicle), and radiation (10Gy) combined with VCX (10Gy+VCX). B) Quantification of TUNEL-positive cells in tail tissues. Experiments represent 6-8 independent repeats, and error bars represent mean ± SEM, *<0.05, and ****P < 0.0001. Scale bar: 50μm. LYVE-1: lymphatic vessel endothelial hyaluronan receptor-1; TUNEL: terminal deoxynucleotidyl transferase dUTP nick end labeling; and DAPI: 4′, 6-diamidino-2-phenylindole.

Supplemental Table 1

	Cytokine/Chemokine Names	Fold Change (48h)	Fold Change (72h)
1	**CCL1/I-309**	0.42	Undetectable
2	**CCL2/MCP-1**	1.15	1.14
3	**MIP-1α/MIP-1β**	Undetectable	Undetectable
4	**CCL5/RANTES**	2.47	2.62
5	**CD40 Ligand/TNFSF5**	Undetectable	Undetectable
6	**Complement Component C5/C5a**	Undetectable	Undetectable
7	**CXCL1/GROα**	1.44	1.57
8	**CXCL10/IP-10**	0.100476154	Undetectable
9	**CXCL11/I-TAC**	Undetectable	Undetectable
10	**CXCL12/SDF-1**	1.06	1.25
11	**G-CSF**	0.76	1.58
12	**GM-CSF**	2.27	2.75
13	**ICAM-1/CD54**	Undetectable	Undetectable
14	**IFN-γ**	Undetectable	Undetectable
15	**IL-1α/IL-1F1**	Undetectable	Undetectable
16	**IL-1β/IL-1F2**	0.030823604	Undetectable
17	**IL-1ra/IL-1F3**	Undetectable	Undetectable
18	**IL-2**	Undetectable	Undetectable
19	**IL-4**	Undetectable	Undetectable
20	**IL-5**	Undetectable	Undetectable
21	**IL-6**	Undetectable	Undetectable
22	**IL-8**	1.3	2.88
23	**IL-10**	Undetectable	Undetectable
24	**IL-12 p70**	0.09	Undetectable
25	**IL-13**	Undetectable	Undetectable
26	**IL-16**	0.12	Undetectable
27	**IL-17A**	Undetectable	Undetectable
28	**IL-17E**	Undetectable	Undetectable
29	**IL-18/IL-1F4**	0.32	Undetectable
30	**IL-21**	0.12	Undetectable
31	**IL-27**	Undetectable	Undetectable
32	**IL-32α**	0.20	Undetectable
33	**MIF**	1.75	1.82
34	**Serpin E1/PAI-1**	1.57	1.53
35	**TNF-α**	Undetectable	Undetectable
36	**TREM-1/CD354**	Undetectable	Undetectable

Supplemental Table 1. Fold increase in cytokines/chemokines following RT in HDLEC. The fold changes in 36 different cytokines and chemokines were determined in HDLECs' supernatants 48 and 72-hours post-RT compared to non-treated cells using a Proteome Profiler Human Cytokine Array Kit. n=2.
