# Supplementary material for: Radiation induces senescence in lymphatic endothelial cells (LECs) and murine tail lymphedema tissue, contributing to lymphedema progression
Source: Cell Death Dis. 2026 Jun 10;17(1):733. doi: 10.1038/s41419-026-08955-z (PMC13478601; doi:10.1038/s41419-026-08955-z)

Supplementary Information

Radiation induces senescence in lymphatic endothelial cells (LECs) and murine tail lymphedema tissue, contributing to lymphedema progression

Samaneh Safarpour et al.
Corresponding author: Spencer B. Gibson, sgibson2@ualberta.ca

This PDF file includes:

Full-length, uncropped original western blots

Figure 5

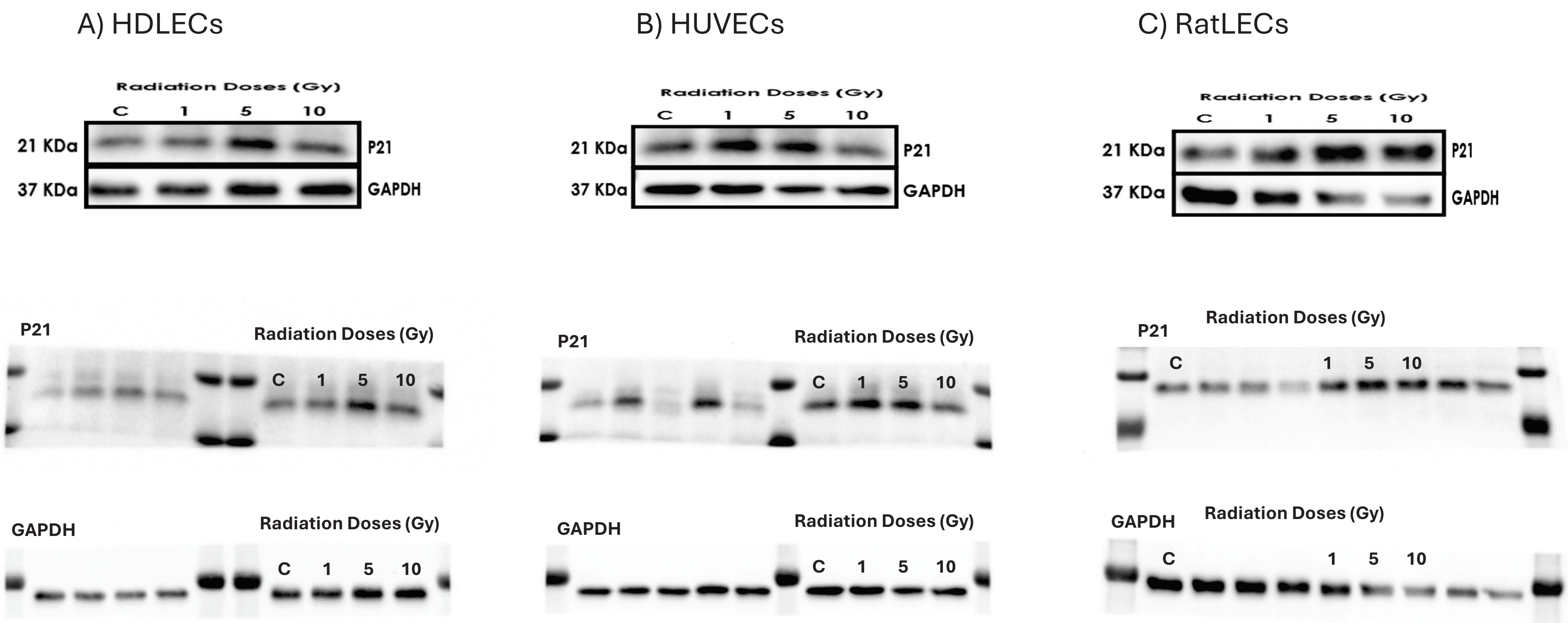
A) HDLECs
Radiation Doses (Gy)
C 1 5 10
21 KDa
P21
37 KDa
GAPDH
P21
Radiation Doses (Gy)
C 1 5 10
GAPDH
Radiation Doses (Gy)
C 1 5 10
B) HUVECs
Radiation Doses (Gy)
C 1 5 10
21 KDa
P21
37 KDa
GAPDH
P21
Radiation Doses (Gy)
C 1 5 10
GAPDH
Radiation Doses (Gy)
C 1 5 10
C) RatLECs
Radiation Doses (Gy)
C 1 5 10
21 KDa
P21
37 KDa
GAPDH
P21
Radiation Doses (Gy)
C 1 5 10
GAPDH
Radiation Doses (Gy)
C 1 5 10

Figure 5

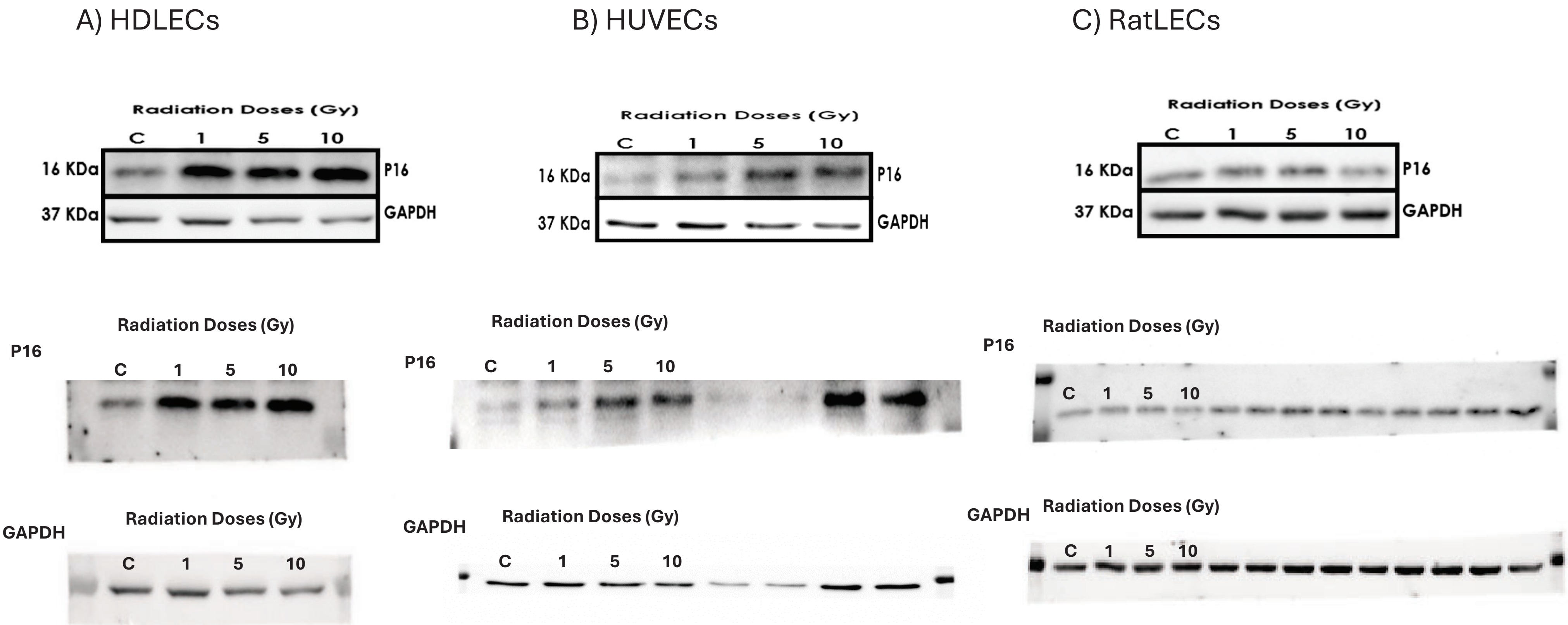
A) HDLECs
B) HUVECs
C) RatLECs
Radiation Doses (Gy)
C
1
5
10
16 KDa
37 KDa
P16
GAPDH

Figure 5

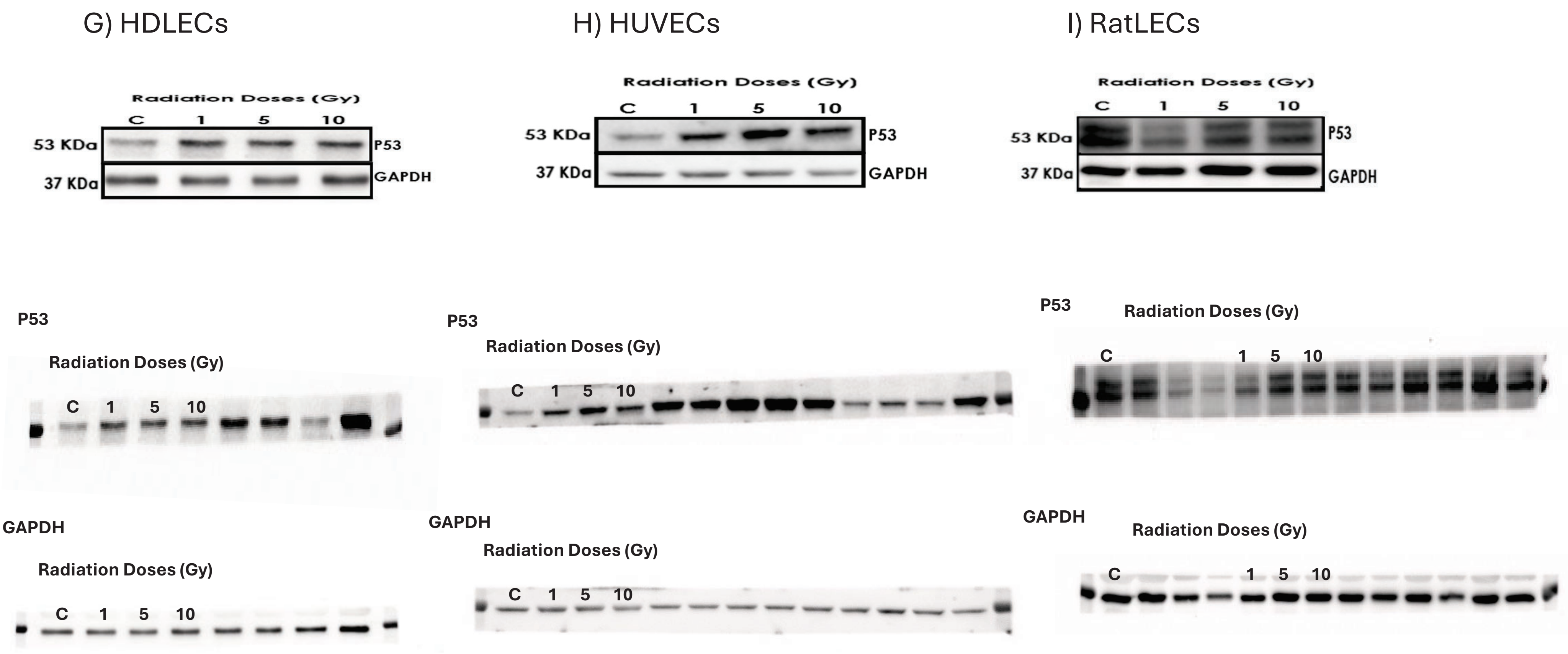
G) HDLECs
H) HUVECs
I) RatLECs
Radiation Doses (Gy)
C 1 5 10
53 KDa
37 KDa
P53
GAPDH

Figure 9

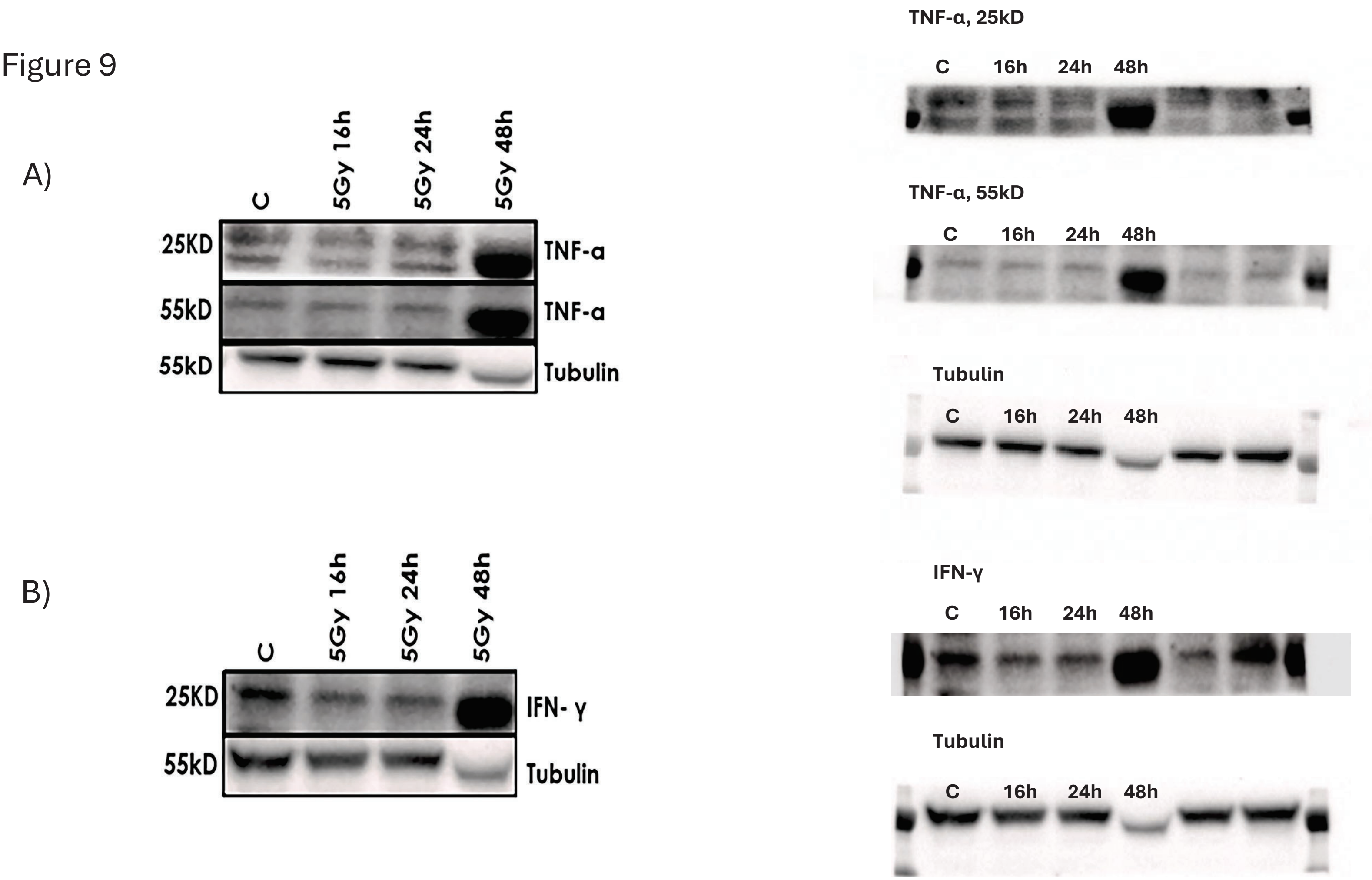
A)
C
5Gy 16h
5Gy 24h
5Gy 48h
25KD
TNF-α
55kD
TNF-α
55kD
Tubulin
B)
C
5Gy 16h
5Gy 24h
5Gy 48h
25KD
IFN- γ
55kD
Tubulin
TNF-α, 25kD
C 16h 24h 48h
TNF-α, 55kD
C 16h 24h 48h
Tubulin
C 16h 24h 48h
IFN-γ
C 16h 24h 48h
Tubulin
C 16h 24h 48h

Figure 10

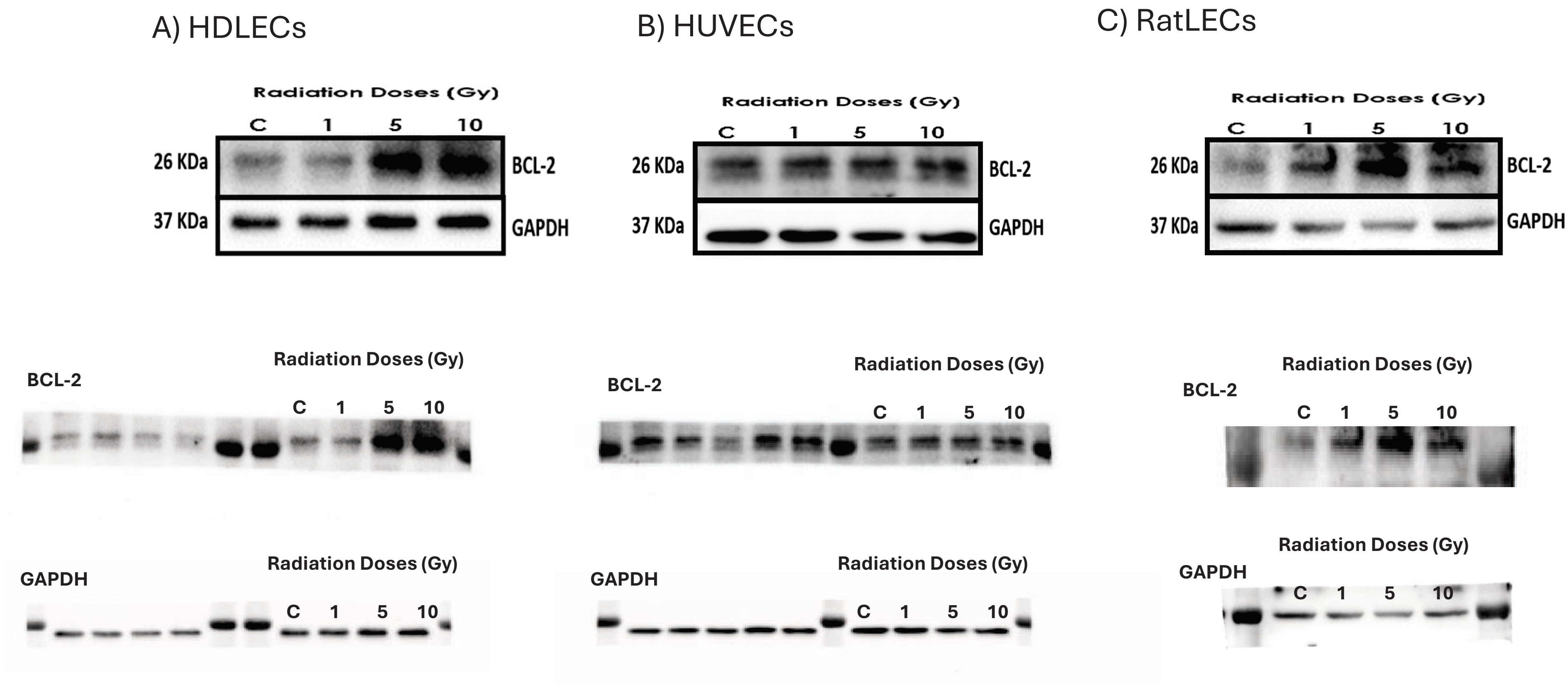

A) HDLECs
B) HUVECs
C) RatLECs
Radiation Doses (Gy)
C 1 5 10
26 KDa
37 KDa
BCL-2
GAPDH

Supplemental Figure 2

G)

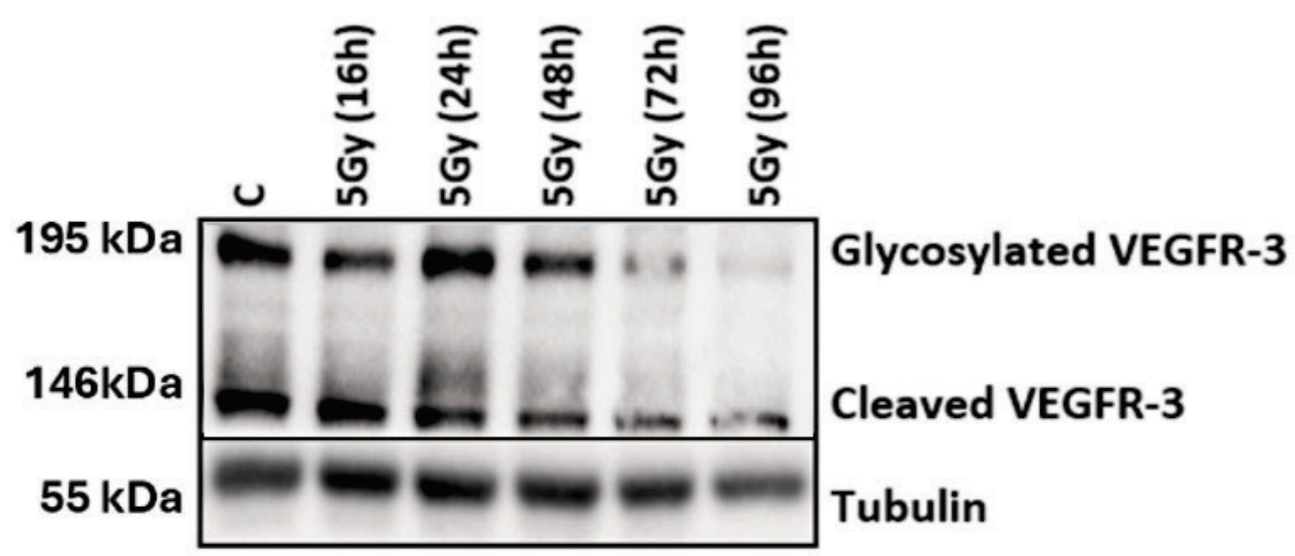

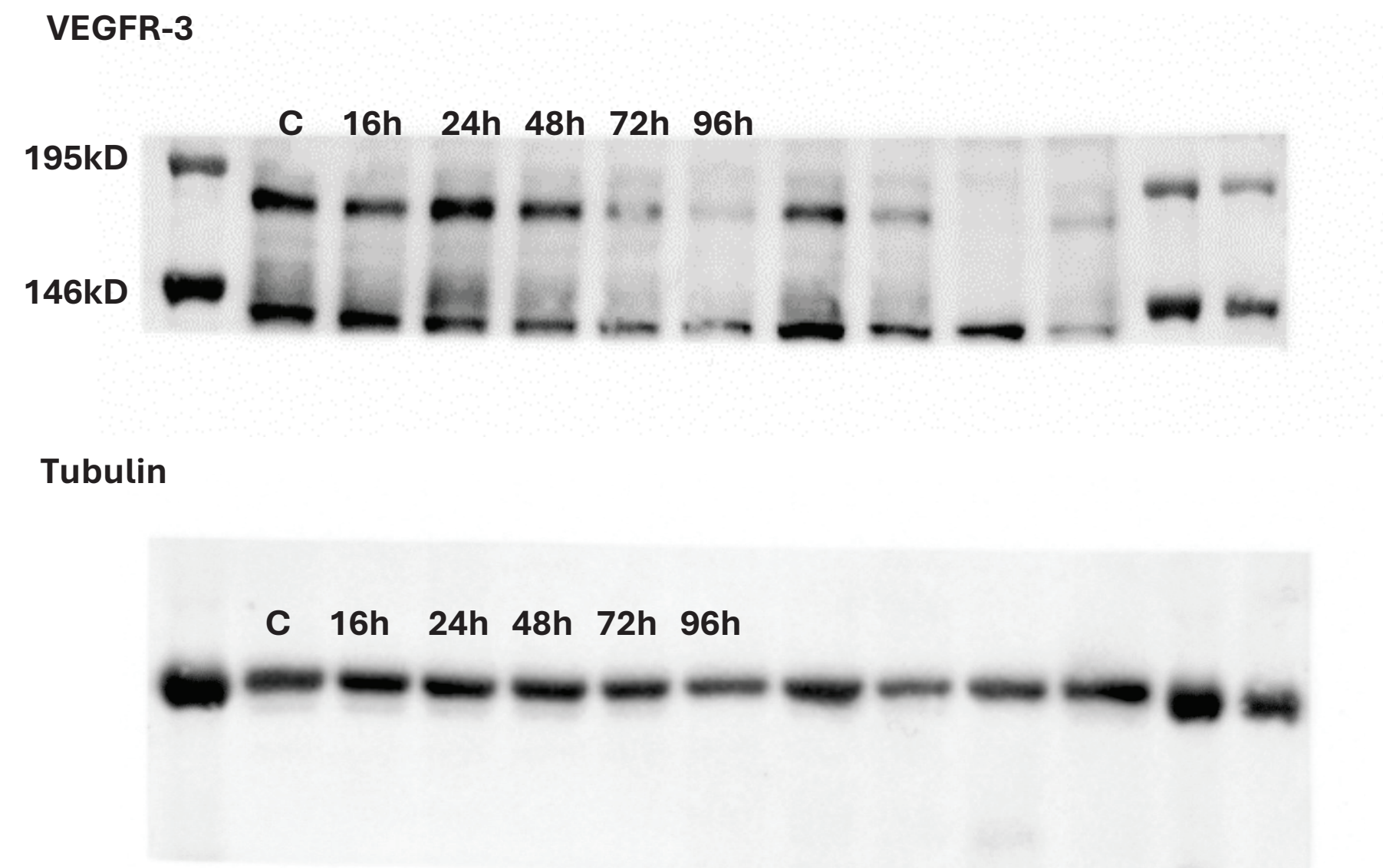

Supplemental Figure 13

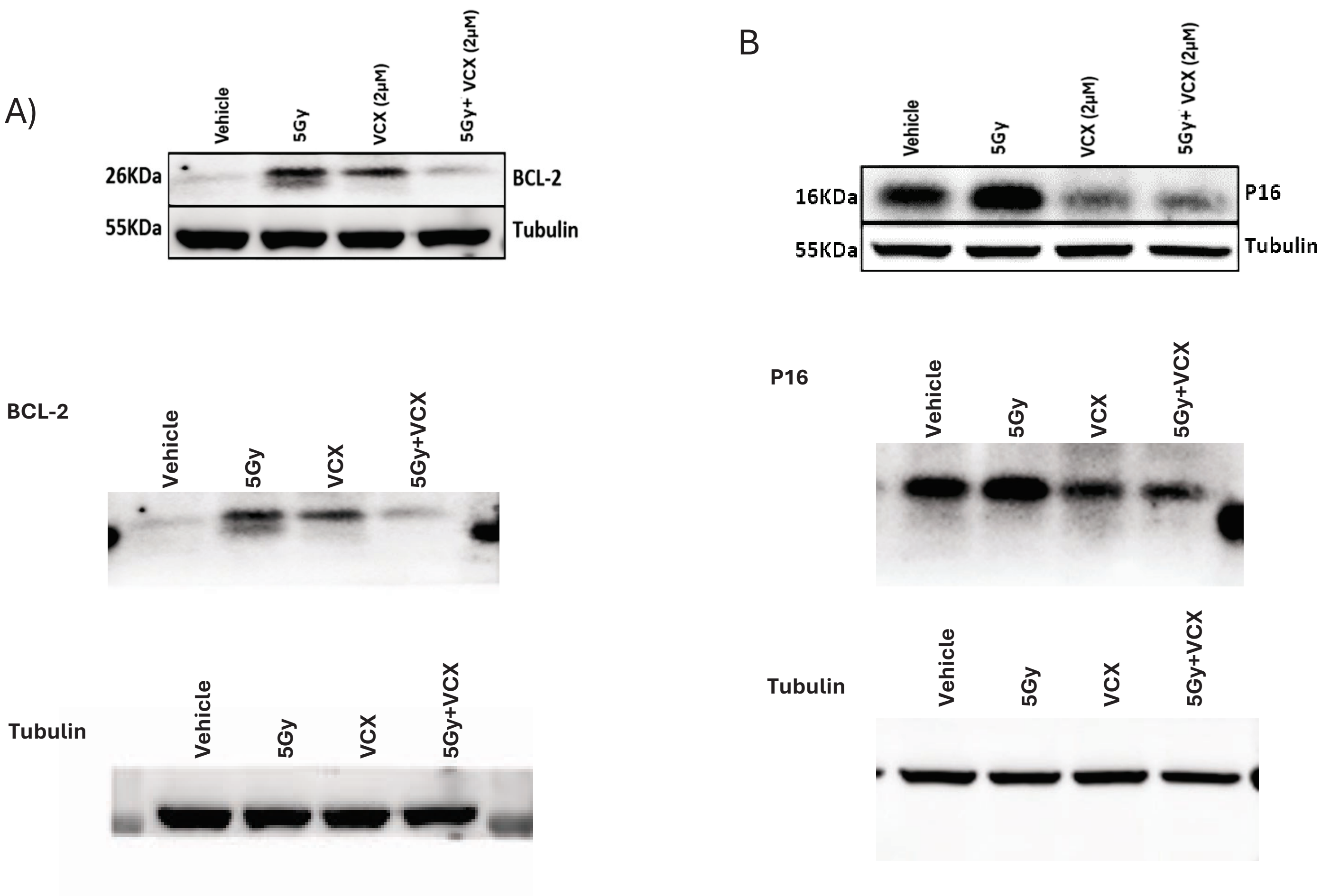

Supplement: Supplementary file 2 [file 41419_2026_8955_MOESM2_ESM.pdf]
